# Inequality in life expectancy losses by education in Finland during COVID-19 pandemic

**DOI:** 10.1186/s12939-026-02827-w

**Published:** 2026-03-28

**Authors:** Dmitri Jdanov, Domantas Jasilionis, Lasse Tarkiainen, Pekka Martikainen

**Affiliations:** 1 Max Planck Institute for Demographic Research, Rostock, Germany; 2 Max Planck – University of Helsinki Center for Social Inequalities in Population Health (MaxHel Center), Helsinki, Finland; 3 Max Planck – University of Helsinki Center for Social Inequalities in Population Health (MaxHel Center), Rostock, Germany; 4 Helsinki Institute for Demography and Population Health, University of Helsinki, Helsinki, Finland

**Keywords:** COVID-19, Life expectancy losses, Inequality, Educational groups

## Abstract

**Background:**

The COVID-19 pandemic has resulted in a rapid increase in mortality in most societies examined. The effects of the pandemic have varied considerably between different populations, time periods and age groups. Studies also report pronounced socioeconomic differentials, with mortality rates being higher in lower socioeconomic groups. However, the evidence about mortality changes across socioeconomic groups is somewhat patchier.

**Data:**

We use annual death counts and population exposures by 5-year age groups for ages 30 + and open age group 100+, sex, and education from the Finnish population register. We also used data on a number of COVID-19 deaths by age and sex published by the Statistical Office of Finland.

**Methods:**

Using the excess mortality approach, we estimate life expectancy losses in Finland in 2020–2024 by education, and quantify the contribution of changes in age-specific mortality to the total changes in differences in life expectancy between the high and low education groups. The abridged life tables for ages 30 + were calculated using observed and counterfactual (Lee-Carter estimates) death rates with open age group 100 + without smoothing. Counterfactual death counts were calculated using observed population estimates and expected death rates assuming no pandemic scenario. We used stepwise decomposition to estimate the contribution of age-specific groups to changes in life expectancy and inequality measures.

**Results:**

We found that life expectancy losses vary considerably by year, sex, and education group. During the first pandemic year 2020, the most affected group was males with an intermediate level of education, showing life expectancy loss of 0.40 years. Interestingly, the corresponding loss among higher educated males was almost the same (0.36 years). Meanwhile, the life expectancy decline in the low education group was only 0.2 years. At the same time, females in all educational groups showed only negligible changes. The situation reversed between 2021 and 2023, with the most significant losses being observed among low-educated males and females in 2022. These education-specific trends contributed to widening life expectancy differences.

**Conclusion:**

The group that benefits more from the health care system in normal circumstances will also be at an advantage in the crisis situation. Our study confirms the importance of combatting socioeconomic inequalities and better protecting vulnerable groups in order to reduce the life expectancy losses at the national level during the mortality crisis.

**Supplementary Information:**

The online version contains supplementary material available at 10.1186/s12939-026-02827-w.

## Background

The COVID-19 pandemic led to substantial mortality increases affecting entire national and subnational populations. However, pandemic-related excess mortality considerably varied across and within global regions and countries [1–4]. Prior research suggests that the effect of the pandemic was more pronounced in the lower socio-economic groups [5–7]. However, most of the available evidence on socio-economic differentials in excess mortality relies on limited data covering only the first years of the pandemic.

The majority of the developed countries experienced life expectancy losses already during the first year of the COVID-19 pandemic. However, in Finland, life expectancy in 2020 remained at about the same level as reported in 2019 [8]. The situation worsened during the following two years (2021 and 2022), showing pronounced life expectancy declines. The recuperation in life expectancy started in 2023, with life expectancy almost returning to the pre-pandemic level. In the Nordic region, a similar dynamic was observed in Norway and Denmark, but not in Sweden [8].

Despite maintaining strong pro-equitable social policies and having a long tradition of monitoring health inequalities, research suggests unfavourable tendencies in mortality inequalities in the Nordic countries [9–14]. Available data show that the COVID-19 pandemic seems to have very uneven effects across different population groups. For example, one of the first Swedish studies relying on data for 2020 confirms that excess mortality due to COVID-19 and other causes of death disproportionally hit those with the lowest education, having low income, and immigrants from low- or middle-income countries [15, 16]. Similar pattern and notable increase in mortality inequalities by socio-economic status were reported in Denmark during 2020–2021 [7]. Evidence, including the last pandemic years (2022 and 2023), is very limited despite some worrying findings about the rapidly worsening situation in pandemic-related losses for the countries experiencing lower levels of excess mortality in the initial phases of the pandemic [17]. This distinct temporal pattern of pandemic-related excess mortality has been particularly pronounced in the Nordic countries. For example, Finland showed three times higher excess mortality - based on linear extrapolation of sex- and age-specific mortality rates of 2010–2019 - in 2022 than observed in 2021 [18].

This study using education as a measure of socio-economic status provides new evidence on changes in education-specific excess mortality and related life expectancy losses in Finland during the pandemic years (2020–2023) and the first post-pandemic year (2024). The objectives of the paper are (1) to estimate pandemic-related losses by educational groups during the whole period of the pandemic; (2) to estimate the influence of trends and initial differences on mortality inequality by educational groups in 2020–2024.

First, in order to quantify the total (direct and indirect) pandemic-related losses within each educational group, the excess mortality framework was applied by comparing the observed and counterfactual (assuming no pandemic) education-specific mortality trends. Second, using demographic decomposition methods, we identified the exact contributions of excess mortality within each age group to the total education-specific losses in life expectancy at age 30. Third, using an innovative contour decomposition method, we also assessed to what extent the life expectancy gap by education in 2022 was driven by (a) pre-pandemic education-specific mortality trends and (b) education-specific mortality differentials during the pandemic period. Finally, we compared the numbers of excess deaths and deaths with COVID-19 as the underlying cause of death on a death certificate.

## Data and methods

### Data

We used annual death counts and population exposures for years 2005–2022 by 5-year age groups for ages 30 + and open age group 100+, sex, and education aggregated from the individual-level data originating from the registers of Statistics Finland (permission TK/1836/07.03.00/2024). In the dataset used for the analysis, 14 cells with male death counts below three and greater than zero (i.e. death counts 1 or 2) were suppressed due to confidentiality reasons (0.000002% of all male person-years). We replaced these missing values with a value of 1.5. Three educational groups are defined as follows: (a) basic education (less than primary, primary or lower secondary education (ISCED 0–2)); (b) intermediate education (upper secondary education (ISCED 3–4)), and 3) high education (tertiary education (ISCED 5–8)). We also used data on the number of deaths with COVID-19 virus infection (ICD-10 codes: U071, U072, U109) as the underlying cause of death by age and sex published by Statistics Finland [19].

Following previous studies, we have chosen to focus on individuals aged 30 years and over, in order to avoid uncertainty related to high educational mobility at younger ages. Administrative and register data, including information from the educational and population registers, are highly complete in Finland and are considered the gold standard today [20].

### Methods

Like the majority of studies on mortality due to COVID-19, we applied the excess mortality approach, which compares the observed education-specific mortality rates to counterfactual (expected) education-specific mortality rates assuming no pandemic scenario (Leon et al. 2020; Beaney et al. 2020; Islam 2022). Following Islam et al. (2021), we estimated annual life expectancy losses using expected values from the short-term mortality forecast. The Lee-Carter model [21] was used to extrapolate annual age-specific death rates that consequently serve as input to estimate counterfactual life expectancy values and death counts during 2020-24. The model was fitted separately by sex and education group using the observed data from 2005 to 2019. We also performed sensitivity analysis for the choice of the reference period. More information on the optimal choice of the reference period might be found elsewhere [22].

The total (national) death rates were calculated as the weighted sum of the educational groups. In addition, the Lee-Carter forecast for the national population was compared with counterfactual death rates derived from specific educational population groups. This comparison revealed a high degree of consistency between the data series.

Education-specific abridged life tables for ages 30 + were calculated using observed and counterfactual (Lee-Carter estimates) age- and education-specific death rates with the last open age interval 100+. Counterfactual (expected) death counts assuming no pandemic scenario were calculated by multiplying observed (official) population exposures and expected death rates. The education-specific excess deaths were calculated as the difference between observed death counts and the counterfactual number of deaths calculated using the observed population and forecasted death rates.

The mortality forecast, i.e. the estimates of counterfactual mortality level, is considered to be the only source of uncertainty. The confidence intervals for counterfactual mortality rates were derived from the Lee-Carter forecast. The Lee-Carter forecast and calculation of respective confidence limits for mortality rates were performed using the R package Demography [23]. We employed Chiang’s formula to estimate confidence intervals for counterfactual life expectancy and related measures (losses, differences, etc.) using the confidence interval for forecasted rates. We calculated confidence intervals for death counts and excess deaths, assuming that the population is unaffected by random fluctuations.

A method of stepwise decomposition of aggregated demographic measures [24] was applied to estimate the exact contributions of excess mortality within age-specific groups to the total life expectancy losses during each pandemic year. This method quantifies the contributions of differences in death rates within each age group (whether positive or negative) to the total difference in life expectancy at age 30 between two population groups. A positive contribution indicates lower mortality in the group in question, which increases the difference, while a negative contribution indicates higher mortality in the first population, which decreases the difference. Finally, the contour decomposition method [25] was used to estimate to what extent the life expectancy gap by education in 2022 (the year with the most severe pandemic impact) and in 2024 (first post-pandemic year) depends on (a) pre-pandemic education-specific mortality trends and (b) observed differences by education-specific mortality during the pandemic period. Contour decomposition is an extension of the stepwise decomposition method, which further splits the age-specific contributions into trend and initial components.

## Results

Before the COVID-19 pandemic, life expectancy by education and the gap between high and basic education groups showed varying trends for both males and females (Table 1 and Supplementary Material Fig. 1S). For males, one may observe a moderate decline in the gap from the peak of 7.5 years in 2008 to roughly 6.5 years during the years preceding the pandemic. Between 2005 and 2019, the female life expectancy gap was generally widening despite some fluctuations from the low of 4.1 years in 2007 to the high of 5.4 years in 2019 (Table 1).

**Table 1:** Life expectancy at age 30 by sex and education, and the difference between high and basic education groups in Finland

Year	Males					Females				
	Basic	Inter mediate	High	Total	Diff.	Basic	Inter mediate	High	Total	Diff.
2005	43.87	47.21	50.89	46.63	7.02	51.25	53.95	55.63	53.05	4.38
2006	44.37	47.35	50.77	46.94	6.40	51.42	54.19	55.84	53.38	4.42
2007	43.95	47.28	51.20	47.00	7.24	51.48	54.08	55.62	53.47	4.14
2008	44.41	47.55	51.88	47.42	7.46	51.34	54.04	56.24	53.58	4.90
2009	44.44	47.71	51.42	47.48	6.98	51.57	54.34	55.77	53.69	4.19
2010	44.83	47.80	51.84	47.74	7.01	51.56	54.14	56.05	53.75	4.49
2011	45.08	48.27	52.08	48.16	7.00	51.59	54.53	56.18	54.07	4.59
2012	45.54	48.41	52.18	48.43	6.64	51.47	54.59	56.29	54.03	4.81
2013	45.62	48.93	52.48	48.67	6.86	51.87	54.51	56.40	54.24	4.53
2014	46.08	48.78	52.56	48.97	6.48	51.67	54.80	56.81	54.31	5.14
2015	46.38	49.36	52.74	49.30	6.36	51.95	54.86	56.83	54.54	4.88
2016	45.97	49.25	52.94	49.23	6.97	51.93	54.96	56.66	54.61	4.72
2017	46.56	49.58	53.09	49.57	6.53	51.94	54.94	56.86	54.70	4.92
2018	46.57	49.54	53.09	49.70	6.52	52.04	54.79	57.02	54.77	4.98
2019	46.72	49.87	53.57	50.05	6.84	51.85	55.07	57.25	55.04	5.40
2020	46.71	49.51	53.37	49.94	6.66	51.91	54.99	57.39	55.06	5.49
2021	46.41	49.64	53.61	49.95	7.21	51.32	55.00	57.32	54.95	5.99
2022	45.93	49.28	53.14	49.52	7.21	50.74	54.27	56.66	54.29	5.92
2023	46.33	49.39	53.55	49.83	7.21	50.92	54.56	57.04	54.69	6.11
2024	47.06	50.03	54.00	50.49	6.94	51.35	55.37	57.61	55.30	6.26

The first year (2020) of the COVID-19 pandemic did not bring any abrupt changes in total and education-specific life expectancy. The total life expectancy remained almost at the same level, whereas males with high and intermediate education saw modest life expectancy decreases (-0.2 and − 0.4 years, respectively). The corresponding changes in total and education-specific female life expectancy were negligible. During the following two pandemic years (2021–2022), the observed gap widened by roughly half a year compared to 2019 among both men and women. The maximal difference was found for 2023 for males and for 2024 for females.

Table 2; Fig. 2S in the Supplementary materials show the total and education-specific life expectancy losses for the pandemic and first post-pandemic years estimated by comparing the observed and counterfactual (assuming no pandemic) mortality. First, we found that the first pandemic year did not bring any substantial losses for females. However, males exhibited notable losses across all educational groups, with the most pronounced losses in the intermediate and high education groups. Strikingly, the males with the lowest level of education experienced the least pronounced losses. Second, educational gradient in male and female life expectancy losses returned to an expected pattern in 2021, showing the most pronounced life expectancy losses occurring in the basic education group and the least pronounced losses in the highly educated group. This divergence in pandemic burden resulted in an increase (approximately by 10%) in the educational gap in life expectancy compared with the previous year. Third, quite similar losses (0.9–1.1 years) were observed for high and intermediate education groups among both males and females in 2022; slightly bigger losses (1.2–1.3 years) occurred among the lowest educated males and females. Thus, despite significant overall increases in mortality in 2022, there was no further growth in the educational life expectancy gap for males. In 2023, there was a modest improvement in life expectancy for both sexes and for all educational groups. However, there has been no improvement in the disparity between the groups. In 2024, the first post-pandemic year, the highest education group almost reached the expected level of life expectancy for both sexes. The educational life expectancy gap for females continued to increase, reaching its maximum at this point.

**Table 2:** Losses in life expectancy (difference between counterfactual and observed life expectancy) at age 30 by education (basic, intermediate, high, total population) with 95% confidential interval

Year	Basic			Intermediate			High			Total		
**Males**												
	Losses	95% CI		Losses	95% CI		Losses	95% CI		Losses	95% CI	
2020	0.19	0.15	0.23	0.50	0.47	0.53	0.36	0.32	0.40	0.32	0.29	0.36
2021	0.68	0.62	0.73	0.53	0.48	0.57	0.28	0.23	0.33	0.51	0.47	0.56
2022	1.33	1.26	1.40	1.05	0.99	1.10	0.92	0.85	0.98	1.14	1.08	1.20
2023	1.10	1.02	1.18	1.09	1.03	1.15	0.67	0.59	0.74	1.03	0.96	1.10
2024	0.55	0.45	0.64	0.61	0.54	0.67	0.37	0.28	0.45	0.56	0.48	0.63
**Females**												
2020	-0.03	-0.09	0.04	0.16	0.13	0.19	-0.04	-0.09	0.00	-0.02	-0.06	0.02
2021	0.59	0.50	0.68	0.21	0.16	0.26	0.13	0.07	0.20	0.21	0.15	0.27
2022	1.20	1.09	1.31	1.01	0.95	1.07	0.89	0.81	0.97	0.98	0.91	1.06
2023	1.05	0.92	1.18	0.79	0.72	0.86	0.61	0.52	0.71	0.70	0.61	0.79
2024	0.64	0.49	0.80	0.04	-0.04	0.12	0.13	0.02	0.24	0.20	0.10	0.30

**Fig. 1:**
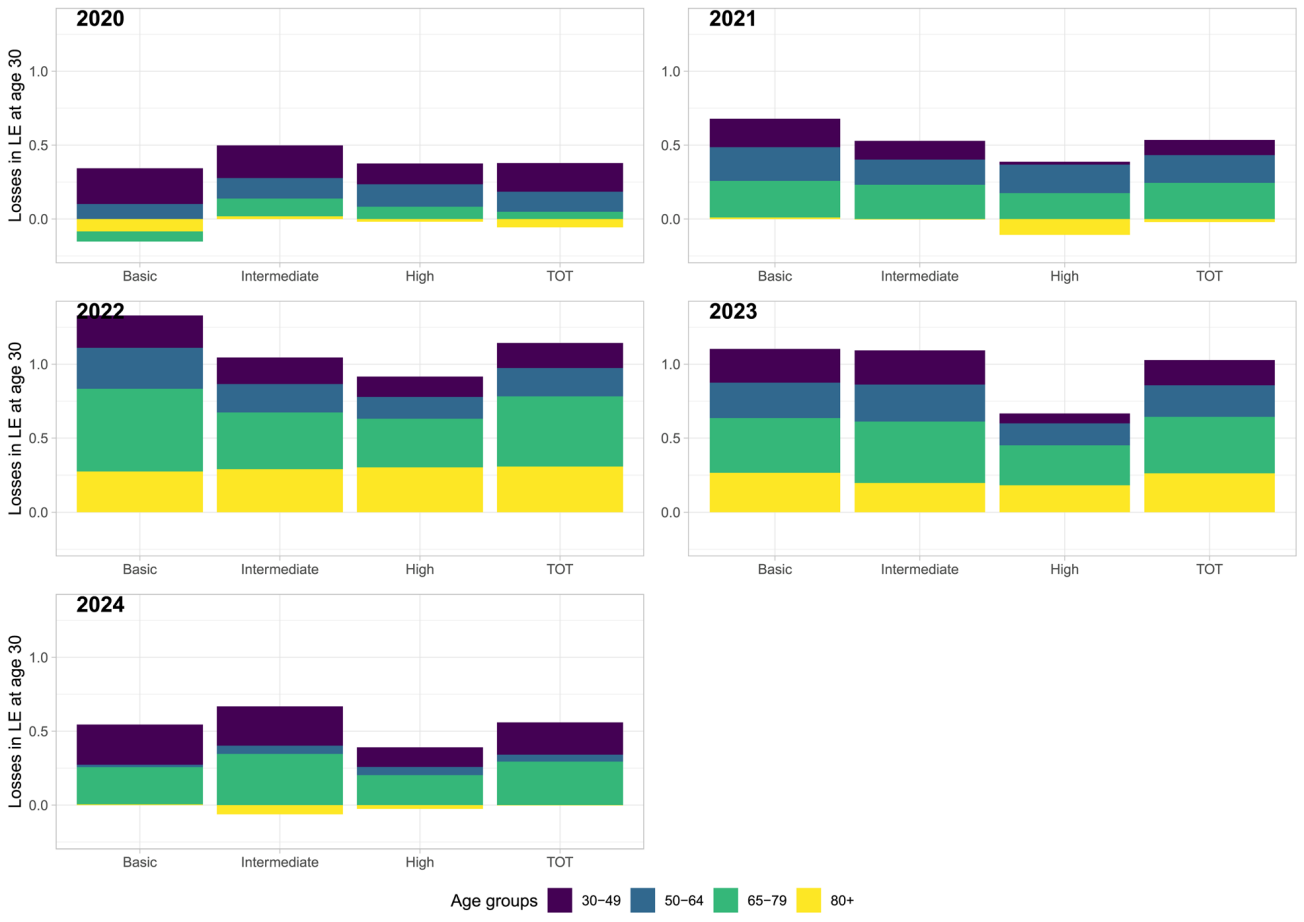
Age-specific contributions to life expectancy losses (difference between counterfactual and observed life expectancy) by education groups and in the total population, males

**Fig. 2:**
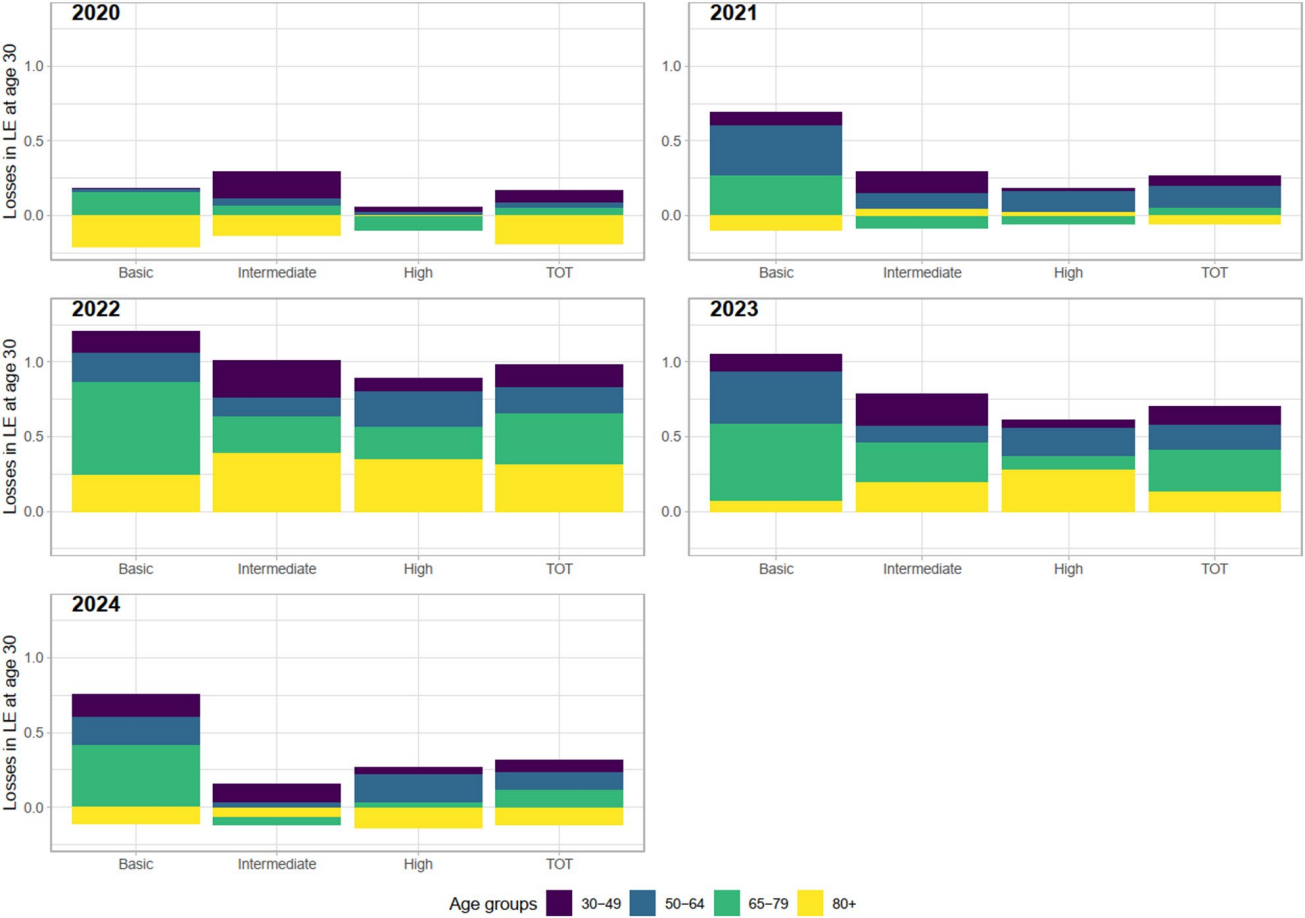
Age-specific contributions to life expectancy losses (difference between counterfactual and observed life expectancy) by education groups and in the total population, females

The age-specific contributions of the differences between the observed and counterfactual (under no pandemic scenario) death rates to the total life expectancy losses show notable variations across educational groups and over time (Figs. 1 and 2). In 2020, total male life expectancy decreases mostly came from increasing death rates at ages 30–49 and 50–64 years. The role of the youngest (30–49 years) age group was particularly pronounced for the least educated males. Interestingly, there was an opposite (negative) effect coming from the age group 80 years and over, suggesting that observed death rates at old ages were lower than predicted under the counterfactual (no pandemic) scenario. Yet this pattern mostly stems from the continuation of improvement in the basic education group, whereas no or only negligible effects can be observed in the high (both sexes) and intermediate (males) education groups. Already in 2021, increased mortality among males aged 65–79 years made the biggest contribution to the total life expectancy losses, and in 2022 and in the following years, the biggest contribution had very clearly shifted to ages 65–79 as well as 80+. Among women, a similar shift took place, with the contribution of older age-groups becoming ever more important. The exact contributions of the two oldest age groups differed by education, with the lowest education groups showing a higher importance of the age group of 65–79 years.

**Fig. 3:**
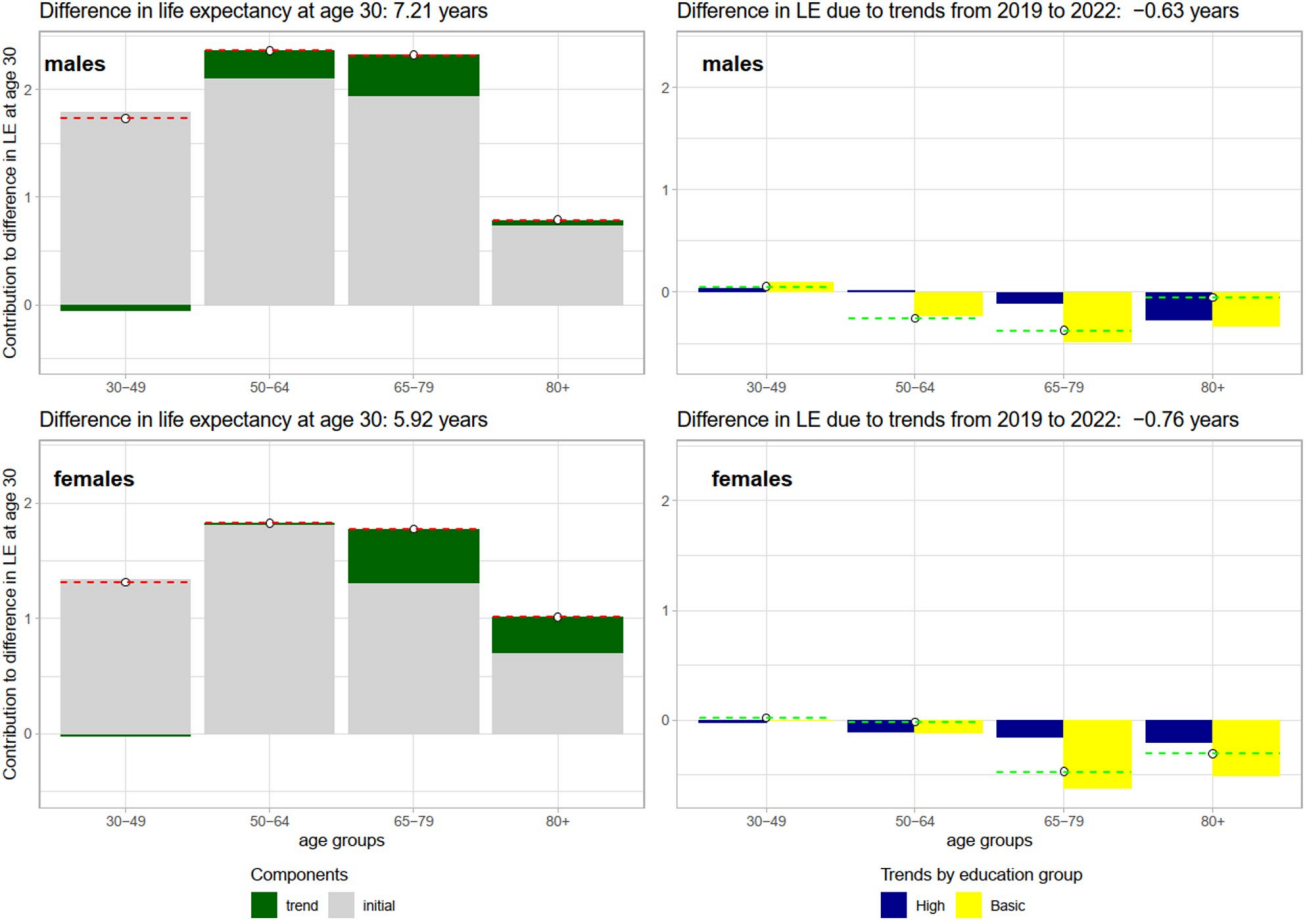
Contour decomposition of the difference in life expectancy at age 30 between high and basic educational groups in 2022

In order to examine the impact of the pre-pandemic education-specific mortality differences on the observed educational gap in life expectancy in 2022 (the year with the highest pandemic losses) and in 2024 (the first post-pandemic year), a contour decomposition was employed. This decomposition quantified the exact age-specific contributions of (a) the initial pre-pandemic mortality conditions (as observed in 2019) and (b) pandemic-related mortality changes (trends) to the total gap in life expectancy at age 30 between the high and basic educational groups in 2022 (Fig. 3) or in 2024 (Fig. 3s). As illustrated in the left panels of Fig. 3, we observe that the total gap in life expectancy is largely attributable to the initial mortality conditions and differentials prevailing prior to the 2019 pandemic. This panel presents the age-specific contributions to the difference in life expectancy, which are additionally decomposed into the trend component (dark green area) and the initial component (light grey area). The contribution of the initial component (mortality differences in the pre-pandemic period) is moderated by the trend component (the temporal change in education specific mortality during the pandemic). For every age group, the sum of the trend and initial components is equal to the total age-specific contributions to the total difference in life expectancy at age 30 (dashed red line). In 2022, the trend contribution at ages 50–79 for males and at age 65 + for females significantly increased the difference in life expectancy between the high and low education groups. Other age groups did not make any significant influence. This means that the negative trend was more pronounced for the least educated group, as shown in the right-hand panel. This panel shows a further decomposition of the trend component (the dark green area on the left-hand panel). The dark blue bars correspond to the age-specific contributions to the trend for the higher-educated group, while the yellow bars represent the respective contributions for the basic-education group. The negative values indicate a decrease in life expectancy since the beginning of the pandemic. The difference in trends between the basic and high educational groups (dashed green line) contributes to the difference in life expectancy as follows. Negative difference in education-specific trends increases the difference in life expectancy, as this means that the negative trends in the basic education group are progressing faster than in the high education group. A positive difference refers to a better performance in the basic education group, which contributes to the decrease in life expectancy gap. The decomposition results also show that the overall life expectancy gap increased moderately as a result of more pronounced increases in mortality after 2019 among those with basic education in the 50–64 and 65–74 age groups for males, and in the 65–79 and 80 + age groups for females. A comparison to the same decomposition for counterfactual life expectancy (assuming no pandemic scenario, as illustrated in Fig. 3S) suggests that the change (trend) component estimated for the same period 2020–2022 produces only a small contribution to the life expectancy gap in 2022. This finding suggests that the observed pandemic-related excess mortality significantly contributed to the increasing educational gap, whereas no pandemic counterfactual scenario suggests only a negligible change.

The contour decomposition of the gap in life expectancy at age 30 between the high and basic educational groups in 2024 (Fig. 3S) demonstrated a comparable dependency on the initial component, i.e. the educational difference in 2019. However, the trend component was only found to be significant among elderly females. This finding suggests that pre-pandemic trends are likely to persist also in the post-pandemic era.

In our final analysis, we make a comparison between the total number of excess deaths and the official number of deaths caused by the novel coronavirus, i.e. with number of deaths with indicated COVID-19 as the cause of death in the death certificate. Despite the fact that this comparison does not contribute to the primary objective of the study, it is necessary in order to establish a clear comparison point with official statistics. Table 3 shows that there were excess deaths across all ages for all educational groups except the oldest age group. Negative excess deaths (i.e., a reduction in the number of deaths compared to the counterfactual level) were observed in the 80-and-over age group in both males and females in 2020 and 2021. This pattern resulted in a negative discrepancy between the number of excess deaths and the number of deaths directly attributed to COVID-19, with the former exceeding the latter during each respective year. The increasing burden of the pandemic in subsequent years led to an increase in excess deaths that could not be directly attributed to the diagnosis of the COVID-19 infection. The registration of deaths from COVID-19 depends on the extent to which the virus is being tested for, which may vary considerably over time and across different groups in the population. Consequently, the results of this comparison should be interpreted with caution. This issue is of a particular significance for the last year (2024), for which excess deaths rather refer to the deviation from pre-pandemic trends than reflecting direct pandemic losses.

**Table 3:** Comparison of COVID-19 deaths and excess deaths. Difference refers to the difference between excess deaths and COVID-19 deaths. Excess deaths are given with 95% confidence intervals in parentheses

Year	Indicator	Age group				
		30–49	50–64	65–79	80+	Total
**Males**						
2020	Excess	180 (154;206)	209 (131;286)	133 (-17;283)	-385 (-503;-266)	137 (-71;345)
	COVID-19	9	27	95	150	281
	Difference	171	182	38	535	-144
	%	95	87	28	-	-106
2021	Excess	93 (56;129)	287 (178;395)	700 (475;925)	-80 (-255;95)	999 (692;1307)
	COVID-19	21	72	221	224	538
	Difference	72	215	479	-304	461
	%	77	75	68	-	46
2022	Excess	163 (118;209)	292 (160;424)	1446 (1156;1736)	1396 (1172;1620)	3298 (2905;3690)
	COVID-19	25	111	718	1418	2272
	Difference	138	181	728	-22	1026
	%	85	62	50	-2	31
2023	Excess	160 (107;213)	306 (156;457)	1199 (844;1554)	958 (695;1220)	2623 (2153;3092)
	COVID-19	6	39	401	818	1264
	Difference	154	267	798	140	1359
	%	96	87	67	15	52
2024	Excess	215 (155;275)	52 (-115;220)	851 (437;1266)	-445 (-750;-140)	673 (129;1218)
	COVID-19	1	17	124	250	392
	Difference	214	35	727	-695	281
	%	100	68	85	-	42
**Females**						
2020	Excess	64 (51;77)	43 (6;80)	131 (53;210)	-960 (-1145;-776)	-722 (-927;-518)
	COVID-19	0	5	47	224	276
	Difference	64	38	84	-1184	-998
	%	100	88	64	-	138
2021	Excess	58 (39;77)	192 (140;244)	136 (16;256)	-434 (-703;-165)	-48 (-347;252)
	COVID-19	9	29	97	277	412
	Difference	49	163	39	-711	-460
	%	85	85	29	-	963
2022	Excess	122 (99;146)	239 (176;302)	883 (726;1040)	1618 (1280;1955)	2862 (2484;3241)
	COVID-19	15	54	380	1619	2068
	Difference	107	185	503	-1	794
	%	88	77	57	0	28
2023	Excess	96 (69;123)	223 (151;295)	767 (572;963)	564 (168;960)	1651 (1203;2099)
	COVID-19	4	13	228	717	962
	Difference	92	210	539	-153	689
	%	96	94	70	-27	42
2024	Excess	72 (41;103)	147 (66;227)	330 (98;563)	-757 (-1214;-300)	-208 (-728;312)
	COVID-19	1	12	54	207	274
	Difference	71	135	276	-964	-482
	%	99	92	84	-	232

Mortality among the elderly showed an inconsistent trend during the period from 2005 to 2019. To test the possible influence of the choice of fitting period, we performed a sensitivity analysis. The results are presented in Supplementary Materials Table 1S. Despite some differences for specific ages, the choice of fitting period does not significantly change the overall results.

Table 2S in the Supplementary Materials provides expected and observed life expectancies by 5-year age groups, educational status, and sex.

## Discussion

The present study employs the excess mortality approach to ascertain life expectancy losses according to educational attainment in Finland through the course of the COVID-19 pandemic during 2020–2023 and the first post-pandemic year. To our knowledge, this study provides the first estimates of life expectancy losses by education in Finland for the whole period of the COVID-19 pandemic. We found that the educational gradient of these losses was not apparent for 2020, but the pattern reversed towards the expected direction in following years. The findings of the study demonstrated that individuals from lower educational backgrounds were particularly vulnerable during the pandemic, but only during 2021–2023. It is evident that throughout 2021–2023, a substantial decline in life expectancy in a group with low educational attainment resulted in a marked increase in inequality between educational groups. Concurrently, the mortality decrease exhibited by the highly educated group after 2022 was found to be superior. The early recovery process was initiated, and in 2024, the trend had almost reached the expected level, i.e. the life expectancy that should have been attained in 2024 according to a counterfactual scenario in which a pandemic had not occurred. The maximum level of inequality for both sexes was not reached in the year with the highest losses (2022), but in the last year of the pandemic (males) or even after the pandemic (females). A number of factors may provide a rationale for the explanation of these trends.

First, it is evident that individuals with a higher level of educational attainment first demonstrated heightened sensitivity to the measures implemented in response to the pandemic. Higher level of trust in science and government, which is more prevalent among the highly educated, is associated with reduced pandemic losses is supported by the evidence [26]. Similar temporal changes were observed at the national level in Europe: while the pandemic reproduced a historical East-West mortality divide with higher excess mortality in Eastern Europe in 2022, there was no such distinct pattern observed in 2020 [26]. The higher excess mortality in socioeconomically disadvantaged Eastern European countries during the second year of the COVID-19 pandemic was partially attributed to lower trust in both government and science and lower vaccination rates [26]. These explanations can also potentially be valid for socio-economically disadvantaged groups [27, 28].

Second, the COVID-19 pandemic in Finland in its initial phase of 2020 was managed by the Finnish Government with a hybrid strategy of both containment and testing & tracking. No hard lockdowns were implemented at any stage of the pandemic, except for brief regional travel restrictions. These measures aimed to keep society and the economy relatively open and succeeded in keeping infections, hospitalizations, and mortality at a low level until the end of 2020. Infections, hospitalizations, and mortality surged in 2021 and particularly in 2022, when vaccination coverage was high and most of the control measures were abandoned [29, 30]. The economic impact of the pandemic was substantial but still not as severe as in many other countries with the unemployment rate increasing during the spring of 2020 from 6.7% to roughly 8% at which level it remained until mid-2021 and declined back to the pre-pandemic level in early 2022. However, the increase in unemployment rate was more pronounced among those with basic and intermediate educational levels [31, 32].

Third, the short-term changes in socioeconomic inequality in mortality have been largely driven by changes in alcohol- and smoking-related mortality in recent decades [14, 33]. These causes are associated with low levels of education and income together with unemployment and other accumulated adversities [34]. A marked decline in alcohol-related mortality has diminished the inequalities between 2008 and 2016, after which the alcohol mortality has stagnated and the inequalities have increased again [33]. Excessive alcohol consumption and particularly smoking more prevalent in the less educated population groups also predict poorer outcomes after COVID-19 infections [35] and may explain part of the increase in mortality inequality.

The middle education group demonstrated a unique pattern at the beginning and end of the pandemic, performing “on average” between the best and worst groups in 2022–23. Initially, they experienced greater losses than other educational groups. During the post-pandemic period, middle-educated males again experienced higher losses, while middle-educated females performed even better than highly-educated females. One potential explanation for this trend is the heterogeneity in composition of this group, particularly among the females [36].

Contour decomposition analyses revealed the nuanced contributions of age-specific differences to mortality inequality during the pandemic, as well as the important role of the corresponding differentials during the pre-pandemic period. First, although working ages (30–64 years) and the age group 65–79 years were the most important for inequality growth during 2020, the contributions shifted towards the oldest age groups in the following years. Though age is the most important risk factor for COVID-19 mortality, increased social mobility and travel at working ages, and consequently higher exposure to the virus [37, 38], have probably played a key role in changing the age pattern of losses in Finland, a country with low losses at the beginning of the pandemic. Second, the initial differences between educational groups observed in 2019 were the key contributors to the life expectancy gap during and after the COVID-19 pandemic, whereas the changes during the pandemic contributed to the widening educational gap for both sexes.

The limitations of our study are related to the excess mortality approach, an approach taken in the majority of COVID-19 studies. First, the total excess mortality attributable to COVID-19 was estimated disregarding other risk factors such as heatwaves, epidemics, and possible disasters other than COVID-19. However, there is no evidence suggesting other similar-scale disasters or crises in Finland during this period. Second, the expected counterfactual mortality assuming no pandemic scenario, as many other forecasts, is affected by uncertainty regarding possible short-term mortality deviations. Nevertheless, the quite narrow confidence intervals suggest that our estimates are not seriously affected by random fluctuations. In addition, the ending of almost a decade-long beneficial development of alcohol related mortality three years before the pandemic may result in a slight overestimation of the hypothetical life expectancy of those with basic education.

A further constraint of the study pertains to the character of the data. We consider fixed educational groups across all ages. However, taking into account the rapid expansion of education, the meaning and composition of these groups may significantly vary across age groups.

Our estimates of the excess deaths for the total Finnish population do not show major differences from those previously published [18]. This close agreement occurs despite important differences in the estimation method in calculating expected deaths under the no pandemic scenario. The estimates of life expectancy losses reported in this study are also in line with estimates for Finland published in several international studies [1, 17, 39, 40]. In general, the total population estimates for Finland show typical temporal trajectories observed in countries that successfully minimized the impact of the first wave of the COVID-19 pandemic. This trend is characterized by a linear and steep increase, with a maximum loss in 2022.

Unlike the majority of other studies on excess mortality during the COVID-19 pandemic, we considered only one measure of socio-economic status - education. The education-specific trends and inequality gradient reported in our study is quite similar to Sweden [41]. Our results are very similar to the estimates of life expectancy losses at age 30 by SES (income quartiles) in Denmark during 2020–2021, with an irregular pattern in 2020 [7].

The comparison of the total excess deaths to the COVID-related deaths based on COVID-19 diagnosis in death certificates might be puzzling because of lower excess deaths than COVID-19 deaths at older ages during the first years of the pandemic. The negative losses in older age groups (80+) during the first two years of the pandemic mean that the number of observed deaths, including those from all causes, was lower than predicted by the model. Given that age is the main risk factor for mortality due to the virus [42], this seems counterintuitive. Nevertheless, this is not an unusual pattern for countries and periods with relatively low pandemic load [43, 44]. Regrettably, this phenomenon is scarcely addressed in the relevant literature. One may assume that individuals in advanced ages receive greater levels of care and experience a significant reduction in their social contacts during periods of pandemic influenza, resulting in a lower overall epidemic burden than would typically be observed. Another possible contributing factor is the potential overestimation of deaths caused by the virus due to issues in the classification of causes of death. However, it is also possible that the model may underestimate the expected number of deaths at very old ages due to the unstable pattern. The sensitivity analysis demonstrated that utilising shorter periods could indeed mediate this effect. However, this does not eliminate the effect entirely, and it does not enhance the overall quality of the model. Consequently, estimates of excess deaths for very old ages, as well as for all other ages, should not be considered as exact numbers in order to avoid a false precision. Nevertheless, this pattern might also be explained by external causes of death, other respiratory diseases (other than COVID-19), and dementia [18] declining particularly in the early stages of the epidemic. These could be thought of as lives saved because of COVID-19. The lower mortality due to non-COVID-19 respiratory diseases most likely reflects a reduced spread of infectious diseases attributable to social distancing [45]. The lower mortality due to dementia may also be associated with a lower risk of death due to non-COVID-19 respiratory diseases, because such diseases as pneumonia are the most common immediate cause of death in individuals with dementia [18].

The global health crisis attributable to the Coronavirus Disease 2019 (COVID-19) pandemic had unprecedented human losses. At the same time, the pandemic has taught important lessons for better preparing for such unexpected health crisis. Our study confirms the importance of combatting socioeconomic inequalities and better protecting vulnerable groups in order to reduce the life expectancy losses at the national level. However, more in-depth studies are needed to identify possible determinants behind the higher excess mortality in lower socioeconomic groups during the COVID-19 pandemic. This evidence is crucial for more effective preventive policies in the context of future health threats related to returning pandemics, climate change, and other disasters.

Statements and Declarations.

## Supplementary Information

Below is the link to the electronic supplementary material.

Supplementary Material 1

## Data Availability

Death counts and population exposures by age, sex, and educational group used and/or analysed during the current study are available upon reasonable request. Access to the data may be granted to those who provide a justified request for research purposes. To request access, please contact the corresponding author. All other datasets are publicly available and are listed in the references.
