# Supplementary material for: Inequality in life expectancy losses by education in Finland during COVID-19 pandemic
Source: Int J Equity Health. 2026 Mar 28;25:90. doi: 10.1186/s12939-026-02827-w (PMC13063482; doi:10.1186/s12939-026-02827-w)
Supplement: Supplementary file 1 — Supplementary Material 1 [file 12939_2026_2827_MOESM1_ESM.docx]

# Supplementary material

*
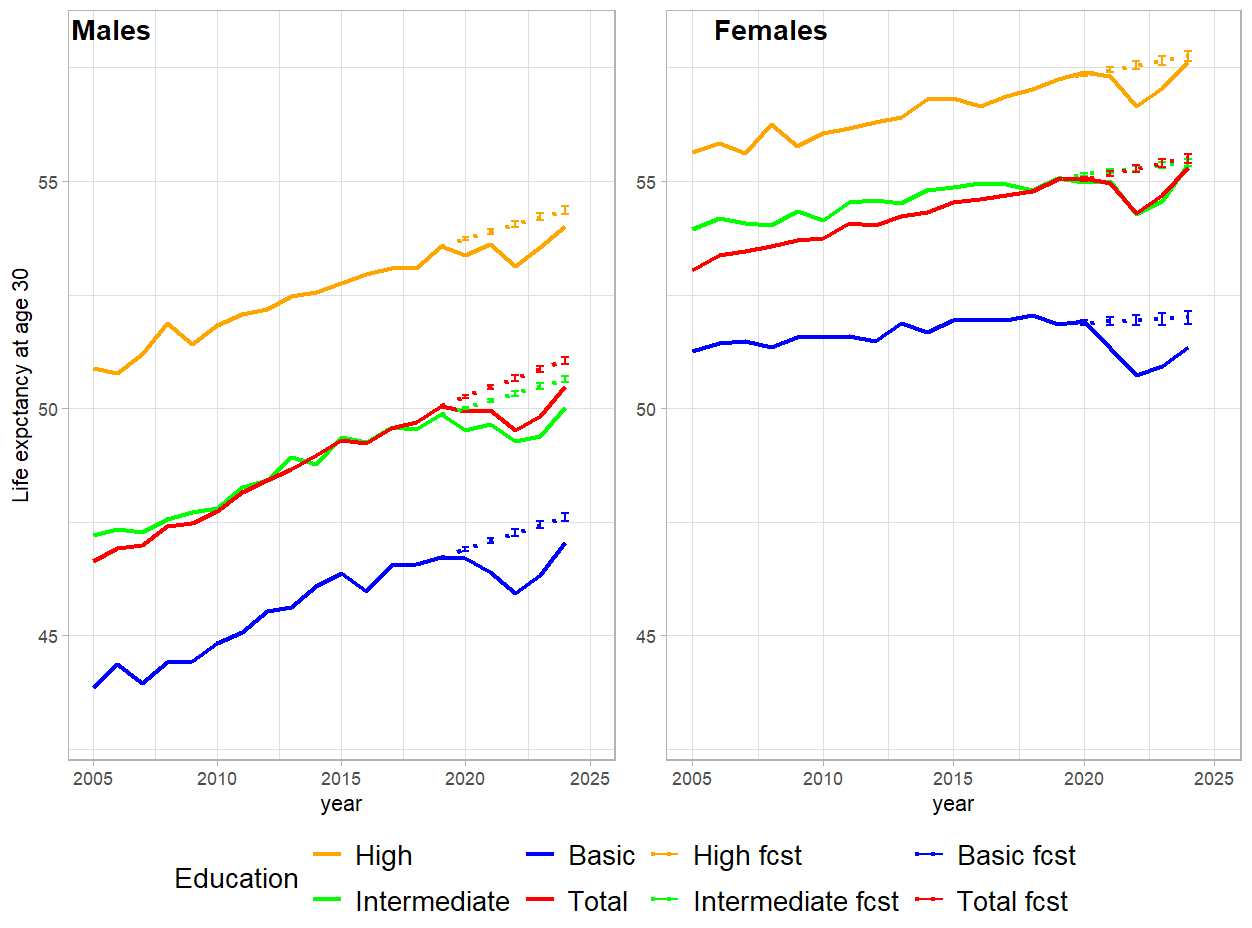
*

Figure 1S. Observed and expected counterfactual (dashed lines with 95% confidence intervals) trends in life expectancy at age 30 by education group


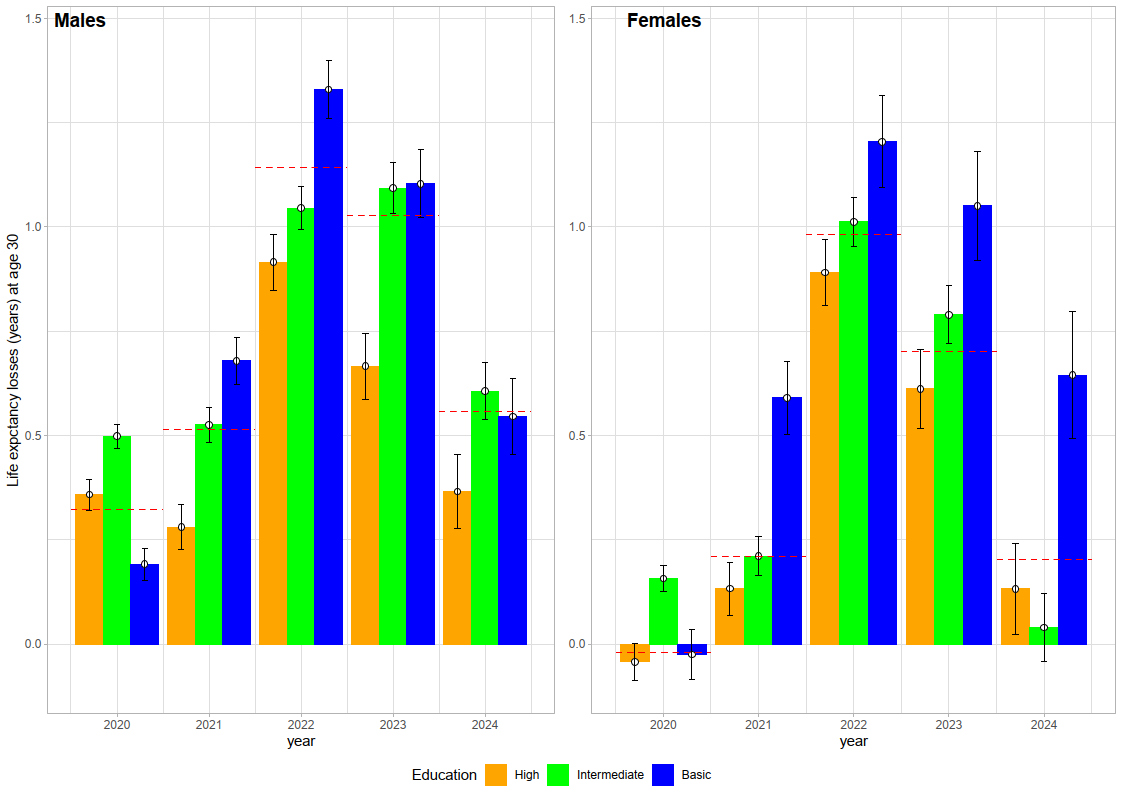


Figure 2S. Life expectancy losses at age 30 by education group in 2020-2021. The red dashed line corresponds to life expectancy losses in total population. The underlying data are in Table 2 in the main text.


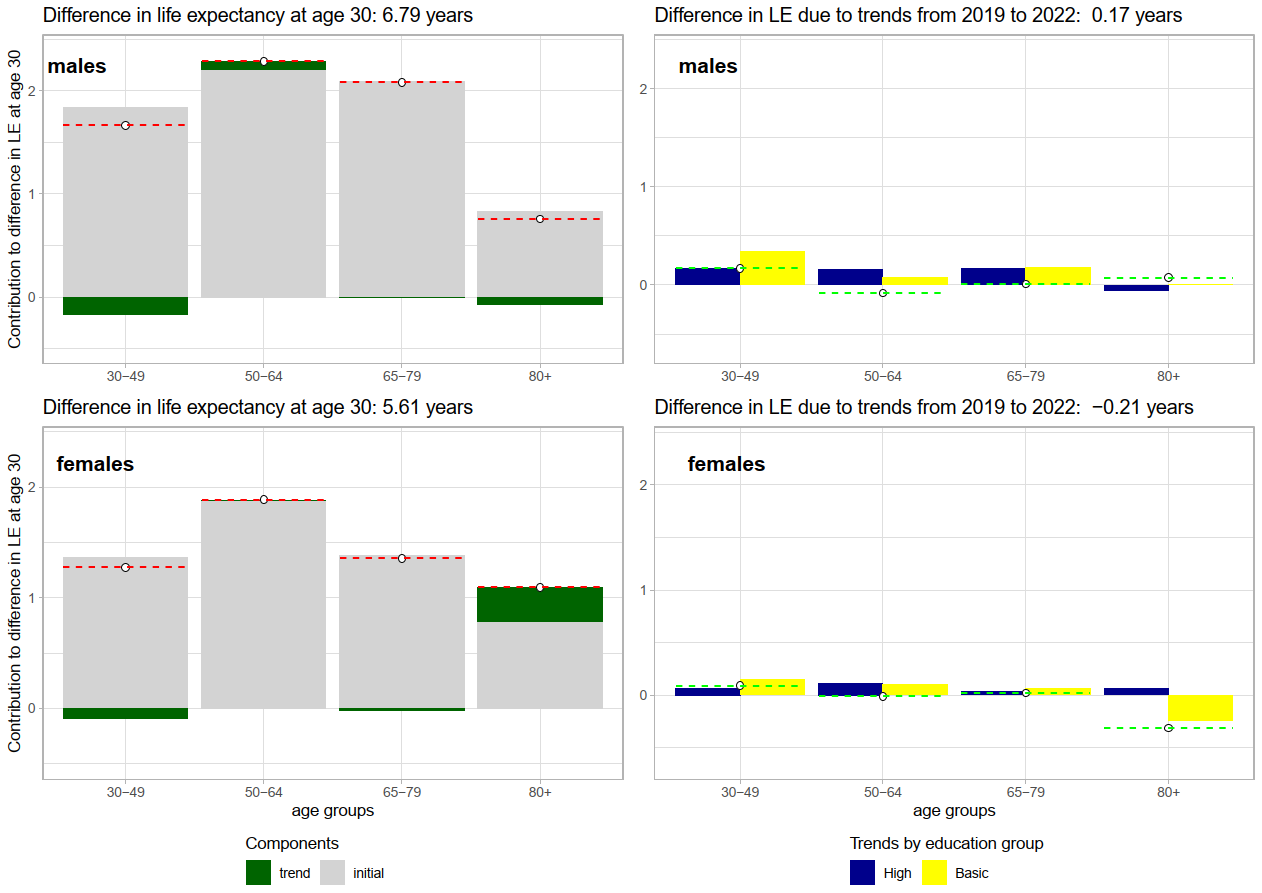


**Figure 3S.** Contour decomposition of difference in life expectance at age 30 between high and basic education group in 2022, no COVID scenario (forecast based on 2005-2019 trends).


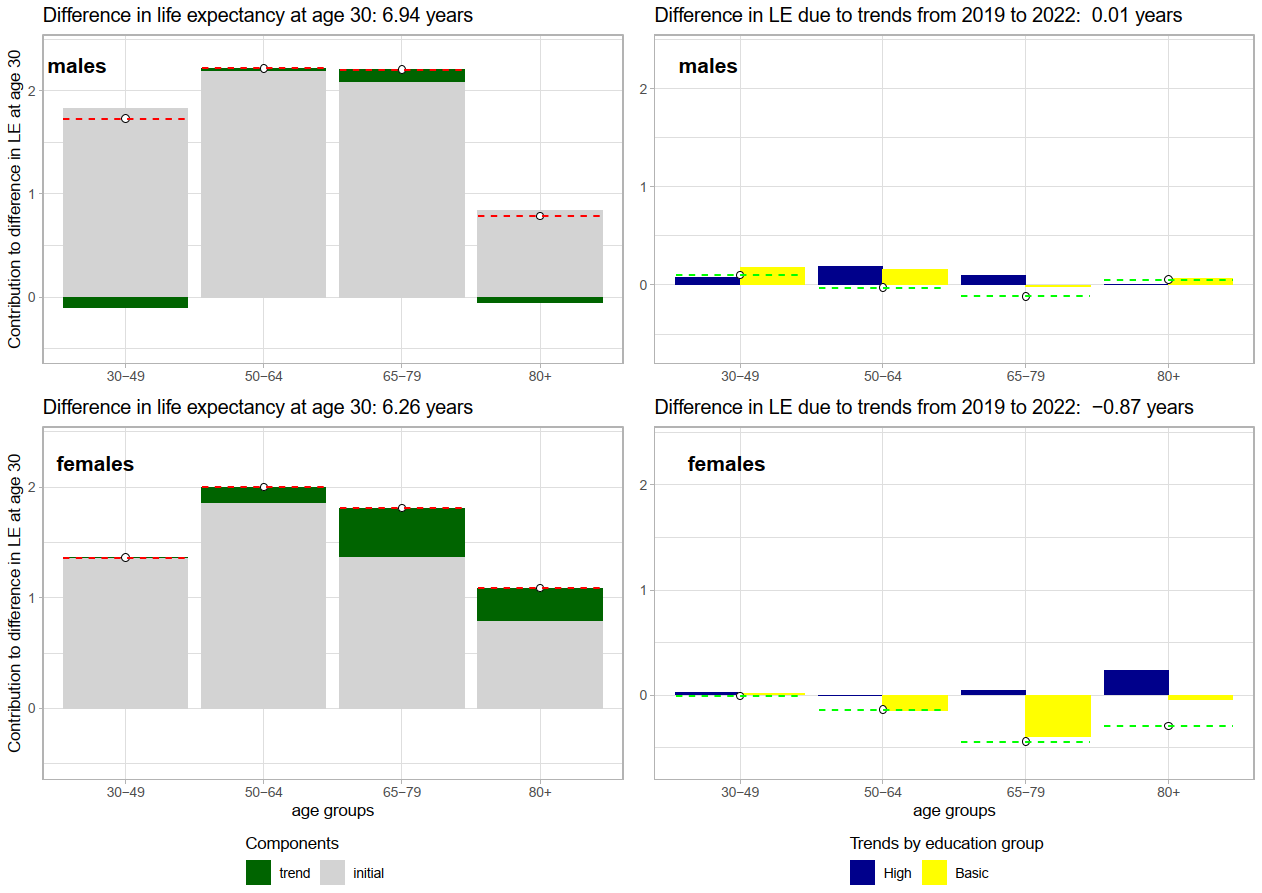


**Figure 4S.** Contour decomposition of difference in life expectance at age 30 between high and basic education group in 2024.

**Table 1S.** Results of sensitivity analysis. Excess deaths are calculated as the difference between the observed and counterfactual (Lee-Carter forecast) estimates. The results for models fitted using the periods 2005–2019 and 2010–2019 are presented. The last column shows the number of deaths officially registered with an underlying cause of death 'Covid-19'. CI are 95% confidence intervals.

|  |  |  | *Fitting period 2005-2019* | | | *Fitting period 2005-2019* | | |  |  |
| --- | --- | --- | --- | --- | --- | --- | --- | --- | --- | --- |
| Year | Sex | Age | Excess | CI low | CI up | Excess | CI low | CI up | Difference | COVID19 |
| 2020 | m | 30 | 180 | 154 | 206 | 172 | 147 | 197 | 8 | 8 |
| 2020 | m | 50 | 209 | 131 | 286 | 248 | 161 | 336 | -40 | 27 |
| 2020 | m | 65 | 133 | -17 | 283 | 86 | -56 | 228 | 46 | 95 |
| 2020 | m | 80 | -385 | -503 | -266 | -201 | -336 | -67 | -184 | 150 |
| 2020 | m | TOT | 137 | -71 | 345 | 305 | 90 | 521 | -169 | 281 |
| 2021 | m | 30 | 93 | 56 | 129 | 88 | 52 | 123 | 5 | 21 |
| 2021 | m | 50 | 287 | 178 | 395 | 343 | 219 | 467 | -56 | 72 |
| 2021 | m | 65 | 700 | 475 | 925 | 653 | 438 | 869 | 47 | 221 |
| 2021 | m | 80 | -80 | -255 | 95 | 158 | -44 | 360 | -238 | 224 |
| 2021 | m | TOT | 999 | 692 | 1307 | 1242 | 920 | 1564 | -242 | 538 |
| 2022 | m | 30 | 163 | 118 | 209 | 161 | 117 | 206 | 2 | 25 |
| 2022 | m | 50 | 292 | 160 | 424 | 364 | 213 | 516 | -72 | 111 |
| 2022 | m | 65 | 1446 | 1156 | 1736 | 1400 | 1119 | 1681 | 46 | 718 |
| 2022 | m | 80 | 1396 | 1172 | 1620 | 1691 | 1430 | 1951 | -295 | 1418 |
| 2022 | m | TOT | 3298 | 2905 | 3690 | 3617 | 3202 | 4031 | -319 | 2272 |
| 2023 | m | 30 | 160 | 107 | 213 | 161 | 108 | 214 | -1 | 6 |
| 2023 | m | 50 | 306 | 156 | 457 | 394 | 219 | 568 | -87 | 39 |
| 2023 | m | 65 | 1199 | 844 | 1554 | 1151 | 805 | 1498 | 47 | 401 |
| 2023 | m | 80 | 958 | 695 | 1220 | 1306 | 998 | 1614 | -349 | 818 |
| 2023 | m | TOT | 2623 | 2153 | 3092 | 3013 | 2514 | 3511 | -390 | 1264 |
| 2024 | m | 30 | 215 | 155 | 275 | 218 | 158 | 279 | -3 | 1 |
| 2024 | m | 50 | 52 | -115 | 220 | 153 | -42 | 348 | -101 | 17 |
| 2024 | m | 65 | 851 | 437 | 1266 | 804 | 395 | 1213 | 47 | 124 |
| 2024 | m | 80 | -445 | -750 | -140 | 58 | -302 | 419 | -504 | 250 |
| 2024 | m | TOT | 673 | 129 | 1218 | 1234 | 652 | 1816 | -560 | 392 |
| 2020 | f | 30 | 64 | 51 | 77 | 13 | -1 | 27 | 51 | 0 |
| 2020 | f | 50 | 43 | 6 | 80 | -51 | -96 | -7 | 94 | 5 |
| 2020 | f | 65 | 131 | 53 | 210 | 158 | 79 | 236 | -26 | 47 |
| 2020 | f | 80 | -960 | -1145 | -776 | -807 | -1015 | -599 | -154 | 224 |
| 2020 | f | TOT | -722 | -927 | -518 | -687 | -914 | -460 | -35 | 276 |
| 2021 | f | 30 | 58 | 39 | 77 | 9 | -11 | 29 | 49 | 9 |
| 2021 | f | 50 | 192 | 140 | 244 | 93 | 30 | 156 | 99 | 29 |
| 2021 | f | 65 | 136 | 16 | 256 | 142 | 20 | 264 | -6 | 97 |
| 2021 | f | 80 | -434 | -703 | -165 | -333 | -641 | -25 | -101 | 277 |
| 2021 | f | TOT | -48 | -347 | 252 | -89 | -427 | 249 | 41 | 412 |
| 2022 | f | 30 | 122 | 99 | 146 | 74 | 49 | 98 | 49 | 15 |
| 2022 | f | 50 | 239 | 176 | 302 | 136 | 58 | 214 | 103 | 54 |
| 2022 | f | 65 | 883 | 726 | 1040 | 865 | 702 | 1028 | 18 | 380 |
| 2022 | f | 80 | 1618 | 1280 | 1955 | 1655 | 1264 | 2047 | -38 | 1619 |
| 2022 | f | TOT | 2862 | 2484 | 3241 | 2730 | 2297 | 3162 | 133 | 2068 |
| 2023 | f | 30 | 96 | 69 | 123 | 45 | 16 | 75 | 51 | 4 |
| 2023 | f | 50 | 223 | 151 | 295 | 116 | 26 | 206 | 107 | 13 |
| 2023 | f | 65 | 767 | 572 | 963 | 720 | 514 | 926 | 47 | 228 |
| 2023 | f | 80 | 564 | 168 | 960 | 537 | 71 | 1002 | 27 | 717 |
| 2023 | f | TOT | 1651 | 1203 | 2099 | 1418 | 900 | 1936 | 233 | 962 |
| 2024 | f | 30 | 72 | 41 | 103 | 17 | -17 | 51 | 55 | 1 |
| 2024 | f | 50 | 147 | 66 | 227 | 36 | -66 | 137 | 111 | 12 |
| 2024 | f | 65 | 330 | 98 | 563 | 250 | 2 | 499 | 80 | 54 |
| 2024 | f | 80 | -757 | -1214 | -300 | -853 | -1397 | -309 | 96 | 207 |
| 2024 | f | TOT | -208 | -728 | 312 | -550 | -1158 | 58 | 342 | 274 |

**Table 2S.** Observed (ex) and counterfactual (ex_noCOVID) life expectancy by age, sex (m=males, f=females), educational group for 2020-2024. Counterfactual life expectancy is given with 95% confidence intervals.

| **Year** | **Sex** | **Educational group** | **Age** | **ex** | **ex_noCOVID** | | | **Difference** | | |
| --- | --- | --- | --- | --- | --- | --- | --- | --- | --- | --- |
|  |  |  |  |  |  | *confidence interval* | |  | *confidence interval* | |
| 2020 | f | Basic | 30 | 51.91 | 51.87 | 51.74 | 52.00 | -0.04 | -0.17 | 0.09 |
| 2020 | f | Basic | 35 | 47.22 | 47.13 | 47.02 | 47.23 | -0.09 | -0.20 | 0.01 |
| 2020 | f | Basic | 40 | 42.53 | 42.48 | 42.37 | 42.59 | -0.05 | -0.16 | 0.06 |
| 2020 | f | Basic | 45 | 37.89 | 37.88 | 37.77 | 37.98 | -0.01 | -0.12 | 0.09 |
| 2020 | f | Basic | 50 | 33.45 | 33.38 | 33.30 | 33.46 | -0.06 | -0.14 | 0.02 |
| 2020 | f | Basic | 55 | 29.10 | 29.16 | 29.08 | 29.25 | 0.07 | -0.01 | 0.15 |
| 2020 | f | Basic | 60 | 25.04 | 25.03 | 24.95 | 25.10 | -0.02 | -0.09 | 0.06 |
| 2020 | f | Basic | 65 | 21.02 | 20.95 | 20.88 | 21.03 | -0.07 | -0.14 | 0.01 |
| 2020 | f | Basic | 70 | 17.11 | 16.97 | 16.89 | 17.04 | -0.15 | -0.22 | -0.07 |
| 2020 | f | Basic | 75 | 13.38 | 13.13 | 13.04 | 13.21 | -0.25 | -0.33 | -0.17 |
| 2020 | f | Basic | 80 | 9.90 | 9.64 | 9.54 | 9.73 | -0.27 | -0.36 | -0.17 |
| 2020 | f | Basic | 85 | 6.91 | 6.65 | 6.56 | 6.75 | -0.26 | -0.36 | -0.16 |
| 2020 | f | Basic | 90 | 4.61 | 4.43 | 4.34 | 4.52 | -0.17 | -0.26 | -0.09 |
| 2020 | f | Basic | 95 | 2.97 | 2.92 | 2.91 | 2.93 | -0.05 | -0.06 | -0.03 |
| 2020 | f | Basic | 100 | 2.20 | 1.92 | 1.86 | 1.99 | -0.27 | -0.34 | -0.20 |
| 2020 | f | High | 30 | 57.39 | 57.37 | 57.35 | 57.40 | -0.02 | -0.04 | 0.01 |
| 2020 | f | High | 35 | 52.45 | 52.41 | 52.39 | 52.44 | -0.04 | -0.06 | -0.01 |
| 2020 | f | High | 40 | 47.51 | 47.48 | 47.45 | 47.50 | -0.03 | -0.06 | -0.01 |
| 2020 | f | High | 45 | 42.61 | 42.56 | 42.53 | 42.58 | -0.05 | -0.08 | -0.03 |
| 2020 | f | High | 50 | 37.76 | 37.71 | 37.68 | 37.73 | -0.06 | -0.08 | -0.03 |
| 2020 | f | High | 55 | 32.96 | 32.91 | 32.89 | 32.94 | -0.04 | -0.07 | -0.02 |
| 2020 | f | High | 60 | 28.24 | 28.22 | 28.19 | 28.24 | -0.03 | -0.05 | 0.00 |
| 2020 | f | High | 65 | 23.65 | 23.61 | 23.59 | 23.64 | -0.04 | -0.06 | -0.01 |
| 2020 | f | High | 70 | 19.18 | 19.20 | 19.18 | 19.22 | 0.02 | 0.00 | 0.04 |
| 2020 | f | High | 75 | 14.96 | 14.95 | 14.93 | 14.98 | 0.00 | -0.03 | 0.02 |
| 2020 | f | High | 80 | 11.01 | 11.01 | 10.99 | 11.03 | 0.00 | -0.02 | 0.03 |
| 2020 | f | High | 85 | 7.69 | 7.65 | 7.62 | 7.67 | -0.04 | -0.06 | -0.02 |
| 2020 | f | High | 90 | 5.04 | 5.01 | 5.00 | 5.02 | -0.03 | -0.05 | -0.02 |
| 2020 | f | High | 95 | 3.17 | 3.29 | 3.28 | 3.30 | 0.12 | 0.11 | 0.13 |
| 2020 | f | High | 100 | 2.03 | 2.51 | 2.45 | 2.56 | 0.48 | 0.42 | 0.53 |
| 2020 | f | Intermediate | 30 | 54.99 | 54.89 | 54.80 | 54.97 | -0.10 | -0.19 | -0.01 |
| 2020 | f | Intermediate | 35 | 50.10 | 50.12 | 50.06 | 50.19 | 0.02 | -0.04 | 0.09 |
| 2020 | f | Intermediate | 40 | 45.25 | 45.26 | 45.20 | 45.33 | 0.01 | -0.06 | 0.08 |
| 2020 | f | Intermediate | 45 | 40.47 | 40.46 | 40.40 | 40.53 | -0.01 | -0.08 | 0.05 |
| 2020 | f | Intermediate | 50 | 35.75 | 35.67 | 35.60 | 35.73 | -0.08 | -0.14 | -0.01 |
| 2020 | f | Intermediate | 55 | 31.11 | 31.07 | 31.00 | 31.13 | -0.05 | -0.11 | 0.02 |
| 2020 | f | Intermediate | 60 | 26.61 | 26.59 | 26.52 | 26.65 | -0.03 | -0.09 | 0.04 |
| 2020 | f | Intermediate | 65 | 22.21 | 22.22 | 22.15 | 22.28 | 0.01 | -0.06 | 0.08 |
| 2020 | f | Intermediate | 70 | 18.06 | 18.02 | 17.95 | 18.09 | -0.04 | -0.11 | 0.03 |
| 2020 | f | Intermediate | 75 | 14.05 | 14.03 | 13.95 | 14.10 | -0.03 | -0.11 | 0.05 |
| 2020 | f | Intermediate | 80 | 10.42 | 10.29 | 10.21 | 10.37 | -0.13 | -0.21 | -0.05 |
| 2020 | f | Intermediate | 85 | 7.18 | 7.19 | 7.09 | 7.28 | 0.01 | -0.09 | 0.10 |
| 2020 | f | Intermediate | 90 | 4.88 | 4.80 | 4.67 | 4.92 | -0.08 | -0.21 | 0.04 |
| 2020 | f | Intermediate | 95 | 3.44 | 3.61 | 3.30 | 3.92 | 0.17 | -0.14 | 0.49 |
| 2020 | f | Intermediate | 100 | 2.50 | 2.35 | 2.26 | 2.43 | -0.16 | -0.24 | -0.07 |
| 2020 | f | TOT | 30 | 55.06 | 54.92 | 54.90 | 54.94 | -0.14 | -0.15 | -0.12 |
| 2020 | f | TOT | 35 | 50.17 | 50.07 | 50.05 | 50.09 | -0.10 | -0.12 | -0.08 |
| 2020 | f | TOT | 40 | 45.29 | 45.19 | 45.17 | 45.21 | -0.10 | -0.12 | -0.08 |
| 2020 | f | TOT | 45 | 40.45 | 40.34 | 40.32 | 40.36 | -0.12 | -0.13 | -0.10 |
| 2020 | f | TOT | 50 | 35.69 | 35.54 | 35.52 | 35.55 | -0.15 | -0.17 | -0.13 |
| 2020 | f | TOT | 55 | 30.99 | 30.87 | 30.85 | 30.89 | -0.12 | -0.13 | -0.10 |
| 2020 | f | TOT | 60 | 26.42 | 26.31 | 26.30 | 26.33 | -0.11 | -0.13 | -0.09 |
| 2020 | f | TOT | 65 | 21.99 | 21.89 | 21.87 | 21.90 | -0.11 | -0.13 | -0.09 |
| 2020 | f | TOT | 70 | 17.79 | 17.66 | 17.64 | 17.67 | -0.13 | -0.15 | -0.12 |
| 2020 | f | TOT | 75 | 13.81 | 13.63 | 13.62 | 13.65 | -0.18 | -0.20 | -0.16 |
| 2020 | f | TOT | 80 | 10.16 | 9.95 | 9.93 | 9.96 | -0.22 | -0.24 | -0.20 |
| 2020 | f | TOT | 85 | 7.05 | 6.86 | 6.84 | 6.87 | -0.19 | -0.21 | -0.17 |
| 2020 | f | TOT | 90 | 4.69 | 4.54 | 4.52 | 4.55 | -0.15 | -0.17 | -0.14 |
| 2020 | f | TOT | 95 | 3.05 | 3.04 | 3.04 | 3.05 | -0.01 | -0.01 | 0.00 |
| 2020 | f | TOT | 100 | 2.22 | 2.03 | 2.03 | 2.03 | -0.18 | -0.18 | -0.18 |
| 2020 | m | Basic | 30 | 46.71 | 46.92 | 46.88 | 46.96 | 0.20 | 0.16 | 0.24 |
| 2020 | m | Basic | 35 | 42.11 | 42.35 | 42.31 | 42.39 | 0.24 | 0.20 | 0.28 |
| 2020 | m | Basic | 40 | 37.60 | 37.79 | 37.75 | 37.83 | 0.19 | 0.15 | 0.23 |
| 2020 | m | Basic | 45 | 33.28 | 33.34 | 33.30 | 33.38 | 0.06 | 0.02 | 0.10 |
| 2020 | m | Basic | 50 | 29.03 | 28.99 | 28.96 | 29.03 | -0.04 | -0.07 | 0.00 |
| 2020 | m | Basic | 55 | 24.98 | 24.93 | 24.90 | 24.96 | -0.05 | -0.08 | -0.02 |
| 2020 | m | Basic | 60 | 21.10 | 20.96 | 20.93 | 20.98 | -0.14 | -0.17 | -0.12 |
| 2020 | m | Basic | 65 | 17.63 | 17.42 | 17.39 | 17.45 | -0.21 | -0.24 | -0.18 |
| 2020 | m | Basic | 70 | 14.19 | 14.07 | 14.04 | 14.09 | -0.12 | -0.15 | -0.09 |
| 2020 | m | Basic | 75 | 11.06 | 10.89 | 10.86 | 10.92 | -0.16 | -0.20 | -0.13 |
| 2020 | m | Basic | 80 | 8.18 | 8.03 | 8.00 | 8.06 | -0.15 | -0.18 | -0.12 |
| 2020 | m | Basic | 85 | 5.79 | 5.61 | 5.59 | 5.63 | -0.19 | -0.21 | -0.16 |
| 2020 | m | Basic | 90 | 3.93 | 3.88 | 3.85 | 3.91 | -0.05 | -0.09 | -0.02 |
| 2020 | m | Basic | 95 | 2.69 | 2.66 | 2.64 | 2.68 | -0.03 | -0.05 | -0.01 |
| 2020 | m | Basic | 100 | 2.02 | 1.93 | 1.92 | 1.94 | -0.09 | -0.10 | -0.08 |
| 2020 | m | High | 30 | 53.37 | 53.74 | 53.72 | 53.76 | 0.37 | 0.34 | 0.39 |
| 2020 | m | High | 35 | 48.46 | 48.81 | 48.78 | 48.83 | 0.34 | 0.32 | 0.37 |
| 2020 | m | High | 40 | 43.57 | 43.92 | 43.90 | 43.95 | 0.35 | 0.32 | 0.37 |
| 2020 | m | High | 45 | 38.74 | 39.07 | 39.05 | 39.10 | 0.33 | 0.31 | 0.36 |
| 2020 | m | High | 50 | 33.99 | 34.26 | 34.24 | 34.28 | 0.27 | 0.25 | 0.29 |
| 2020 | m | High | 55 | 29.35 | 29.55 | 29.53 | 29.58 | 0.21 | 0.19 | 0.23 |
| 2020 | m | High | 60 | 24.89 | 25.05 | 25.03 | 25.07 | 0.16 | 0.13 | 0.18 |
| 2020 | m | High | 65 | 20.56 | 20.69 | 20.67 | 20.71 | 0.13 | 0.11 | 0.15 |
| 2020 | m | High | 70 | 16.48 | 16.52 | 16.49 | 16.54 | 0.03 | 0.01 | 0.06 |
| 2020 | m | High | 75 | 12.63 | 12.74 | 12.72 | 12.76 | 0.11 | 0.09 | 0.13 |
| 2020 | m | High | 80 | 9.25 | 9.29 | 9.26 | 9.31 | 0.04 | 0.02 | 0.06 |
| 2020 | m | High | 85 | 6.44 | 6.36 | 6.34 | 6.38 | -0.08 | -0.10 | -0.06 |
| 2020 | m | High | 90 | 4.18 | 4.02 | 4.01 | 4.04 | -0.15 | -0.17 | -0.14 |
| 2020 | m | High | 95 | 2.77 | 2.59 | 2.54 | 2.64 | -0.18 | -0.23 | -0.13 |
| 2020 | m | High | 100 | 1.41 | 3.12 | 3.02 | 3.22 | 1.71 | 1.61 | 1.80 |
| 2020 | m | Intermediate | 30 | 49.51 | 50.05 | 50.01 | 50.09 | 0.54 | 0.50 | 0.58 |
| 2020 | m | Intermediate | 35 | 44.74 | 45.25 | 45.21 | 45.29 | 0.51 | 0.47 | 0.55 |
| 2020 | m | Intermediate | 40 | 39.99 | 40.49 | 40.45 | 40.52 | 0.49 | 0.46 | 0.53 |
| 2020 | m | Intermediate | 45 | 35.38 | 35.79 | 35.75 | 35.82 | 0.41 | 0.37 | 0.44 |
| 2020 | m | Intermediate | 50 | 30.86 | 31.19 | 31.15 | 31.22 | 0.32 | 0.29 | 0.36 |
| 2020 | m | Intermediate | 55 | 26.47 | 26.73 | 26.69 | 26.76 | 0.26 | 0.22 | 0.29 |
| 2020 | m | Intermediate | 60 | 22.28 | 22.50 | 22.47 | 22.54 | 0.23 | 0.20 | 0.26 |
| 2020 | m | Intermediate | 65 | 18.43 | 18.58 | 18.55 | 18.61 | 0.15 | 0.13 | 0.18 |
| 2020 | m | Intermediate | 70 | 14.78 | 14.84 | 14.82 | 14.87 | 0.06 | 0.04 | 0.09 |
| 2020 | m | Intermediate | 75 | 11.43 | 11.47 | 11.44 | 11.50 | 0.04 | 0.01 | 0.06 |
| 2020 | m | Intermediate | 80 | 8.41 | 8.45 | 8.42 | 8.48 | 0.03 | 0.00 | 0.07 |
| 2020 | m | Intermediate | 85 | 6.06 | 5.77 | 5.74 | 5.80 | -0.29 | -0.32 | -0.26 |
| 2020 | m | Intermediate | 90 | 4.18 | 3.81 | 3.75 | 3.86 | -0.37 | -0.43 | -0.31 |
| 2020 | m | Intermediate | 95 | 2.75 | 2.96 | 2.88 | 3.03 | 0.21 | 0.13 | 0.29 |
| 2020 | m | Intermediate | 100 | 2.02 | 1.92 | 1.91 | 1.94 | -0.10 | -0.11 | -0.08 |
| 2020 | m | TOT | 30 | 49.94 | 50.28 | 50.26 | 50.30 | 0.34 | 0.32 | 0.36 |
| 2020 | m | TOT | 35 | 45.16 | 45.48 | 45.46 | 45.50 | 0.32 | 0.30 | 0.34 |
| 2020 | m | TOT | 40 | 40.41 | 40.72 | 40.70 | 40.73 | 0.31 | 0.29 | 0.33 |
| 2020 | m | TOT | 45 | 35.76 | 36.00 | 35.98 | 36.02 | 0.24 | 0.22 | 0.26 |
| 2020 | m | TOT | 50 | 31.21 | 31.37 | 31.35 | 31.39 | 0.16 | 0.14 | 0.18 |
| 2020 | m | TOT | 55 | 26.79 | 26.89 | 26.88 | 26.91 | 0.11 | 0.09 | 0.13 |
| 2020 | m | TOT | 60 | 22.56 | 22.62 | 22.60 | 22.64 | 0.06 | 0.04 | 0.08 |
| 2020 | m | TOT | 65 | 18.65 | 18.65 | 18.63 | 18.66 | 0.00 | -0.02 | 0.02 |
| 2020 | m | TOT | 70 | 14.93 | 14.89 | 14.87 | 14.90 | -0.04 | -0.06 | -0.03 |
| 2020 | m | TOT | 75 | 11.52 | 11.47 | 11.45 | 11.49 | -0.05 | -0.07 | -0.03 |
| 2020 | m | TOT | 80 | 8.45 | 8.39 | 8.37 | 8.41 | -0.06 | -0.08 | -0.04 |
| 2020 | m | TOT | 85 | 5.97 | 5.79 | 5.78 | 5.80 | -0.18 | -0.19 | -0.16 |
| 2020 | m | TOT | 90 | 4.01 | 3.90 | 3.89 | 3.91 | -0.12 | -0.13 | -0.11 |
| 2020 | m | TOT | 95 | 2.71 | 2.69 | 2.68 | 2.69 | -0.02 | -0.03 | -0.02 |
| 2020 | m | TOT | 100 | 1.85 | 2.10 | 2.09 | 2.10 | 0.24 | 0.24 | 0.25 |
| 2021 | f | Basic | 30 | 51.32 | 51.88 | 51.68 | 52.09 | 0.56 | 0.36 | 0.76 |
| 2021 | f | Basic | 35 | 46.53 | 47.13 | 46.96 | 47.29 | 0.60 | 0.44 | 0.76 |
| 2021 | f | Basic | 40 | 41.94 | 42.48 | 42.32 | 42.64 | 0.54 | 0.38 | 0.70 |
| 2021 | f | Basic | 45 | 37.45 | 37.89 | 37.73 | 38.04 | 0.44 | 0.28 | 0.59 |
| 2021 | f | Basic | 50 | 32.90 | 33.38 | 33.26 | 33.50 | 0.47 | 0.36 | 0.59 |
| 2021 | f | Basic | 55 | 28.61 | 29.16 | 29.04 | 29.28 | 0.55 | 0.42 | 0.67 |
| 2021 | f | Basic | 60 | 24.70 | 25.03 | 24.92 | 25.14 | 0.33 | 0.21 | 0.44 |
| 2021 | f | Basic | 65 | 20.80 | 20.96 | 20.85 | 21.08 | 0.17 | 0.06 | 0.28 |
| 2021 | f | Basic | 70 | 16.90 | 16.99 | 16.88 | 17.10 | 0.09 | -0.03 | 0.20 |
| 2021 | f | Basic | 75 | 13.22 | 13.15 | 13.03 | 13.27 | -0.07 | -0.19 | 0.05 |
| 2021 | f | Basic | 80 | 9.77 | 9.67 | 9.53 | 9.80 | -0.10 | -0.24 | 0.03 |
| 2021 | f | Basic | 85 | 6.85 | 6.68 | 6.53 | 6.82 | -0.17 | -0.31 | -0.03 |
| 2021 | f | Basic | 90 | 4.53 | 4.44 | 4.32 | 4.57 | -0.09 | -0.21 | 0.04 |
| 2021 | f | Basic | 95 | 3.04 | 2.92 | 2.90 | 2.94 | -0.12 | -0.14 | -0.10 |
| 2021 | f | Basic | 100 | 2.03 | 1.91 | 1.81 | 2.01 | -0.13 | -0.22 | -0.03 |
| 2021 | f | High | 30 | 57.32 | 57.50 | 57.46 | 57.53 | 0.18 | 0.14 | 0.22 |
| 2021 | f | High | 35 | 52.36 | 52.53 | 52.50 | 52.57 | 0.18 | 0.14 | 0.21 |
| 2021 | f | High | 40 | 47.41 | 47.59 | 47.56 | 47.63 | 0.19 | 0.15 | 0.22 |
| 2021 | f | High | 45 | 42.51 | 42.67 | 42.64 | 42.71 | 0.17 | 0.13 | 0.20 |
| 2021 | f | High | 50 | 37.67 | 37.82 | 37.78 | 37.85 | 0.15 | 0.11 | 0.19 |
| 2021 | f | High | 55 | 32.88 | 33.02 | 32.98 | 33.05 | 0.14 | 0.10 | 0.17 |
| 2021 | f | High | 60 | 28.21 | 28.31 | 28.28 | 28.35 | 0.11 | 0.07 | 0.14 |
| 2021 | f | High | 65 | 23.65 | 23.70 | 23.67 | 23.74 | 0.06 | 0.02 | 0.09 |
| 2021 | f | High | 70 | 19.19 | 19.28 | 19.25 | 19.32 | 0.09 | 0.06 | 0.13 |
| 2021 | f | High | 75 | 14.93 | 15.03 | 14.99 | 15.06 | 0.10 | 0.06 | 0.13 |
| 2021 | f | High | 80 | 11.03 | 11.07 | 11.03 | 11.10 | 0.04 | 0.00 | 0.08 |
| 2021 | f | High | 85 | 7.82 | 7.69 | 7.65 | 7.72 | -0.13 | -0.17 | -0.10 |
| 2021 | f | High | 90 | 5.19 | 5.03 | 5.01 | 5.04 | -0.16 | -0.18 | -0.14 |
| 2021 | f | High | 95 | 3.43 | 3.30 | 3.28 | 3.32 | -0.13 | -0.15 | -0.12 |
| 2021 | f | High | 100 | 1.77 | 2.60 | 2.52 | 2.68 | 0.82 | 0.74 | 0.91 |
| 2021 | f | Intermediate | 30 | 55.00 | 54.86 | 54.73 | 54.99 | -0.14 | -0.27 | -0.01 |
| 2021 | f | Intermediate | 35 | 50.12 | 50.06 | 49.96 | 50.16 | -0.06 | -0.16 | 0.04 |
| 2021 | f | Intermediate | 40 | 45.27 | 45.20 | 45.10 | 45.30 | -0.07 | -0.17 | 0.03 |
| 2021 | f | Intermediate | 45 | 40.44 | 40.40 | 40.30 | 40.50 | -0.05 | -0.15 | 0.05 |
| 2021 | f | Intermediate | 50 | 35.71 | 35.62 | 35.52 | 35.71 | -0.09 | -0.19 | 0.00 |
| 2021 | f | Intermediate | 55 | 31.11 | 31.01 | 30.91 | 31.11 | -0.09 | -0.19 | 0.00 |
| 2021 | f | Intermediate | 60 | 26.58 | 26.52 | 26.42 | 26.62 | -0.06 | -0.16 | 0.04 |
| 2021 | f | Intermediate | 65 | 22.21 | 22.16 | 22.05 | 22.26 | -0.06 | -0.16 | 0.05 |
| 2021 | f | Intermediate | 70 | 18.01 | 17.96 | 17.85 | 18.06 | -0.06 | -0.17 | 0.05 |
| 2021 | f | Intermediate | 75 | 13.97 | 13.95 | 13.84 | 14.07 | -0.02 | -0.13 | 0.10 |
| 2021 | f | Intermediate | 80 | 10.22 | 10.24 | 10.12 | 10.36 | 0.02 | -0.10 | 0.13 |
| 2021 | f | Intermediate | 85 | 7.12 | 7.13 | 6.99 | 7.28 | 0.02 | -0.13 | 0.16 |
| 2021 | f | Intermediate | 90 | 4.73 | 4.76 | 4.57 | 4.95 | 0.03 | -0.16 | 0.21 |
| 2021 | f | Intermediate | 95 | 3.07 | 3.48 | 3.00 | 3.96 | 0.41 | -0.06 | 0.89 |
| 2021 | f | Intermediate | 100 | 1.60 | 2.21 | 2.09 | 2.33 | 0.61 | 0.49 | 0.73 |
| 2021 | f | TOT | 30 | 54.95 | 55.02 | 54.99 | 55.05 | 0.07 | 0.05 | 0.10 |
| 2021 | f | TOT | 35 | 50.04 | 50.15 | 50.12 | 50.17 | 0.11 | 0.08 | 0.13 |
| 2021 | f | TOT | 40 | 45.17 | 45.27 | 45.24 | 45.30 | 0.10 | 0.08 | 0.13 |
| 2021 | f | TOT | 45 | 40.33 | 40.42 | 40.39 | 40.45 | 0.09 | 0.06 | 0.12 |
| 2021 | f | TOT | 50 | 35.55 | 35.62 | 35.59 | 35.64 | 0.07 | 0.04 | 0.09 |
| 2021 | f | TOT | 55 | 30.88 | 30.95 | 30.92 | 30.97 | 0.07 | 0.04 | 0.09 |
| 2021 | f | TOT | 60 | 26.34 | 26.38 | 26.36 | 26.41 | 0.05 | 0.02 | 0.07 |
| 2021 | f | TOT | 65 | 21.94 | 21.94 | 21.92 | 21.97 | 0.01 | -0.02 | 0.03 |
| 2021 | f | TOT | 70 | 17.71 | 17.70 | 17.68 | 17.73 | -0.01 | -0.03 | 0.02 |
| 2021 | f | TOT | 75 | 13.72 | 13.68 | 13.65 | 13.70 | -0.05 | -0.07 | -0.02 |
| 2021 | f | TOT | 80 | 10.05 | 9.98 | 9.96 | 10.01 | -0.07 | -0.09 | -0.04 |
| 2021 | f | TOT | 85 | 7.01 | 6.88 | 6.85 | 6.90 | -0.13 | -0.16 | -0.11 |
| 2021 | f | TOT | 90 | 4.63 | 4.55 | 4.53 | 4.57 | -0.08 | -0.11 | -0.06 |
| 2021 | f | TOT | 95 | 3.08 | 3.03 | 3.02 | 3.04 | -0.06 | -0.07 | -0.04 |
| 2021 | f | TOT | 100 | 1.93 | 2.00 | 2.00 | 2.00 | 0.07 | 0.07 | 0.07 |
| 2021 | m | Basic | 30 | 46.41 | 47.10 | 47.05 | 47.16 | 0.70 | 0.64 | 0.76 |
| 2021 | m | Basic | 35 | 41.85 | 42.53 | 42.47 | 42.59 | 0.68 | 0.62 | 0.74 |
| 2021 | m | Basic | 40 | 37.30 | 37.96 | 37.90 | 38.01 | 0.66 | 0.60 | 0.72 |
| 2021 | m | Basic | 45 | 32.82 | 33.49 | 33.44 | 33.54 | 0.67 | 0.61 | 0.72 |
| 2021 | m | Basic | 50 | 28.58 | 29.12 | 29.07 | 29.17 | 0.54 | 0.49 | 0.59 |
| 2021 | m | Basic | 55 | 24.60 | 25.04 | 24.99 | 25.09 | 0.44 | 0.39 | 0.49 |
| 2021 | m | Basic | 60 | 20.73 | 21.04 | 21.00 | 21.07 | 0.31 | 0.27 | 0.34 |
| 2021 | m | Basic | 65 | 17.20 | 17.50 | 17.46 | 17.54 | 0.30 | 0.26 | 0.34 |
| 2021 | m | Basic | 70 | 13.91 | 14.14 | 14.10 | 14.18 | 0.23 | 0.19 | 0.28 |
| 2021 | m | Basic | 75 | 10.88 | 10.96 | 10.91 | 11.01 | 0.08 | 0.04 | 0.13 |
| 2021 | m | Basic | 80 | 8.04 | 8.08 | 8.04 | 8.13 | 0.05 | 0.00 | 0.09 |
| 2021 | m | Basic | 85 | 5.67 | 5.64 | 5.60 | 5.67 | -0.04 | -0.07 | 0.00 |
| 2021 | m | Basic | 90 | 3.85 | 3.91 | 3.86 | 3.96 | 0.06 | 0.01 | 0.11 |
| 2021 | m | Basic | 95 | 3.00 | 2.67 | 2.64 | 2.70 | -0.33 | -0.36 | -0.30 |
| 2021 | m | Basic | 100 | 2.47 | 1.95 | 1.93 | 1.96 | -0.53 | -0.54 | -0.51 |
| 2021 | m | High | 30 | 53.61 | 53.91 | 53.87 | 53.94 | 0.29 | 0.26 | 0.33 |
| 2021 | m | High | 35 | 48.70 | 48.97 | 48.94 | 49.01 | 0.27 | 0.24 | 0.31 |
| 2021 | m | High | 40 | 43.74 | 44.09 | 44.05 | 44.12 | 0.35 | 0.31 | 0.38 |
| 2021 | m | High | 45 | 38.87 | 39.24 | 39.20 | 39.27 | 0.36 | 0.33 | 0.40 |
| 2021 | m | High | 50 | 34.09 | 34.41 | 34.38 | 34.45 | 0.32 | 0.29 | 0.36 |
| 2021 | m | High | 55 | 29.44 | 29.69 | 29.66 | 29.73 | 0.25 | 0.22 | 0.28 |
| 2021 | m | High | 60 | 24.96 | 25.18 | 25.15 | 25.22 | 0.23 | 0.19 | 0.26 |
| 2021 | m | High | 65 | 20.66 | 20.80 | 20.77 | 20.84 | 0.14 | 0.11 | 0.18 |
| 2021 | m | High | 70 | 16.55 | 16.60 | 16.57 | 16.63 | 0.05 | 0.02 | 0.08 |
| 2021 | m | High | 75 | 12.80 | 12.82 | 12.78 | 12.85 | 0.02 | -0.02 | 0.05 |
| 2021 | m | High | 80 | 9.42 | 9.34 | 9.31 | 9.38 | -0.08 | -0.11 | -0.05 |
| 2021 | m | High | 85 | 6.37 | 6.38 | 6.35 | 6.41 | 0.01 | -0.02 | 0.04 |
| 2021 | m | High | 90 | 4.13 | 4.00 | 3.98 | 4.03 | -0.13 | -0.15 | -0.10 |
| 2021 | m | High | 95 | 2.93 | 2.55 | 2.48 | 2.63 | -0.38 | -0.45 | -0.30 |
| 2021 | m | High | 100 | 3.53 | 3.40 | 3.26 | 3.55 | -0.12 | -0.27 | 0.03 |
| 2021 | m | Intermediate | 30 | 49.64 | 50.24 | 50.19 | 50.30 | 0.60 | 0.55 | 0.65 |
| 2021 | m | Intermediate | 35 | 44.87 | 45.44 | 45.38 | 45.49 | 0.56 | 0.51 | 0.62 |
| 2021 | m | Intermediate | 40 | 40.11 | 40.66 | 40.61 | 40.72 | 0.55 | 0.50 | 0.60 |
| 2021 | m | Intermediate | 45 | 35.46 | 35.95 | 35.90 | 36.01 | 0.49 | 0.44 | 0.55 |
| 2021 | m | Intermediate | 50 | 30.87 | 31.34 | 31.29 | 31.39 | 0.47 | 0.42 | 0.53 |
| 2021 | m | Intermediate | 55 | 26.42 | 26.86 | 26.81 | 26.91 | 0.44 | 0.39 | 0.49 |
| 2021 | m | Intermediate | 60 | 22.27 | 22.61 | 22.56 | 22.65 | 0.34 | 0.29 | 0.39 |
| 2021 | m | Intermediate | 65 | 18.40 | 18.66 | 18.62 | 18.71 | 0.26 | 0.22 | 0.31 |
| 2021 | m | Intermediate | 70 | 14.82 | 14.89 | 14.85 | 14.93 | 0.07 | 0.03 | 0.11 |
| 2021 | m | Intermediate | 75 | 11.57 | 11.51 | 11.47 | 11.55 | -0.06 | -0.10 | -0.02 |
| 2021 | m | Intermediate | 80 | 8.48 | 8.48 | 8.43 | 8.53 | 0.00 | -0.05 | 0.04 |
| 2021 | m | Intermediate | 85 | 5.92 | 5.76 | 5.72 | 5.81 | -0.16 | -0.21 | -0.12 |
| 2021 | m | Intermediate | 90 | 3.90 | 3.78 | 3.69 | 3.86 | -0.13 | -0.21 | -0.04 |
| 2021 | m | Intermediate | 95 | 2.58 | 3.01 | 2.90 | 3.13 | 0.43 | 0.32 | 0.55 |
| 2021 | m | Intermediate | 100 | 1.59 | 1.98 | 1.96 | 2.01 | 0.39 | 0.37 | 0.42 |
| 2021 | m | TOT | 30 | 49.95 | 50.50 | 50.48 | 50.53 | 0.55 | 0.52 | 0.58 |
| 2021 | m | TOT | 35 | 45.18 | 45.70 | 45.67 | 45.73 | 0.52 | 0.49 | 0.55 |
| 2021 | m | TOT | 40 | 40.39 | 40.93 | 40.90 | 40.96 | 0.53 | 0.51 | 0.56 |
| 2021 | m | TOT | 45 | 35.69 | 36.21 | 36.18 | 36.24 | 0.52 | 0.49 | 0.55 |
| 2021 | m | TOT | 50 | 31.09 | 31.56 | 31.53 | 31.59 | 0.47 | 0.44 | 0.50 |
| 2021 | m | TOT | 55 | 26.66 | 27.07 | 27.04 | 27.10 | 0.41 | 0.38 | 0.44 |
| 2021 | m | TOT | 60 | 22.44 | 22.77 | 22.75 | 22.80 | 0.33 | 0.30 | 0.36 |
| 2021 | m | TOT | 65 | 18.51 | 18.78 | 18.75 | 18.80 | 0.27 | 0.24 | 0.30 |
| 2021 | m | TOT | 70 | 14.84 | 14.98 | 14.96 | 15.01 | 0.14 | 0.12 | 0.17 |
| 2021 | m | TOT | 75 | 11.53 | 11.56 | 11.53 | 11.59 | 0.03 | 0.01 | 0.06 |
| 2021 | m | TOT | 80 | 8.45 | 8.46 | 8.43 | 8.48 | 0.01 | -0.02 | 0.03 |
| 2021 | m | TOT | 85 | 5.88 | 5.82 | 5.80 | 5.84 | -0.05 | -0.07 | -0.04 |
| 2021 | m | TOT | 90 | 3.93 | 3.91 | 3.89 | 3.92 | -0.03 | -0.04 | -0.01 |
| 2021 | m | TOT | 95 | 2.99 | 2.69 | 2.69 | 2.70 | -0.30 | -0.31 | -0.29 |
| 2021 | m | TOT | 100 | 2.44 | 2.13 | 2.12 | 2.13 | -0.32 | -0.32 | -0.31 |
| 2022 | f | Basic | 30 | 50.74 | 51.90 | 51.62 | 52.18 | 1.16 | 0.88 | 1.44 |
| 2022 | f | Basic | 35 | 45.94 | 47.12 | 46.91 | 47.34 | 1.18 | 0.97 | 1.40 |
| 2022 | f | Basic | 40 | 41.36 | 42.48 | 42.27 | 42.70 | 1.12 | 0.90 | 1.33 |
| 2022 | f | Basic | 45 | 36.67 | 37.90 | 37.70 | 38.10 | 1.23 | 1.02 | 1.43 |
| 2022 | f | Basic | 50 | 32.33 | 33.37 | 33.22 | 33.52 | 1.04 | 0.89 | 1.19 |
| 2022 | f | Basic | 55 | 28.04 | 29.15 | 29.00 | 29.31 | 1.11 | 0.95 | 1.26 |
| 2022 | f | Basic | 60 | 23.96 | 25.04 | 24.89 | 25.18 | 1.07 | 0.93 | 1.22 |
| 2022 | f | Basic | 65 | 20.03 | 20.98 | 20.83 | 21.12 | 0.95 | 0.80 | 1.09 |
| 2022 | f | Basic | 70 | 16.36 | 17.01 | 16.86 | 17.15 | 0.65 | 0.51 | 0.79 |
| 2022 | f | Basic | 75 | 12.75 | 13.17 | 13.02 | 13.33 | 0.43 | 0.27 | 0.58 |
| 2022 | f | Basic | 80 | 9.28 | 9.70 | 9.52 | 9.87 | 0.42 | 0.24 | 0.59 |
| 2022 | f | Basic | 85 | 6.41 | 6.70 | 6.52 | 6.88 | 0.29 | 0.11 | 0.47 |
| 2022 | f | Basic | 90 | 4.20 | 4.45 | 4.29 | 4.61 | 0.25 | 0.09 | 0.41 |
| 2022 | f | Basic | 95 | 2.72 | 2.91 | 2.89 | 2.94 | 0.19 | 0.17 | 0.22 |
| 2022 | f | Basic | 100 | 1.69 | 1.89 | 1.77 | 2.02 | 0.20 | 0.08 | 0.33 |
| 2022 | f | High | 30 | 56.66 | 57.62 | 57.57 | 57.66 | 0.96 | 0.91 | 1.01 |
| 2022 | f | High | 35 | 51.69 | 52.65 | 52.61 | 52.70 | 0.96 | 0.92 | 1.01 |
| 2022 | f | High | 40 | 46.78 | 47.71 | 47.67 | 47.76 | 0.93 | 0.89 | 0.98 |
| 2022 | f | High | 45 | 41.89 | 42.79 | 42.74 | 42.83 | 0.90 | 0.85 | 0.94 |
| 2022 | f | High | 50 | 37.06 | 37.93 | 37.88 | 37.97 | 0.87 | 0.82 | 0.91 |
| 2022 | f | High | 55 | 32.30 | 33.12 | 33.08 | 33.17 | 0.82 | 0.78 | 0.87 |
| 2022 | f | High | 60 | 27.63 | 28.41 | 28.37 | 28.46 | 0.78 | 0.73 | 0.82 |
| 2022 | f | High | 65 | 23.09 | 23.79 | 23.75 | 23.84 | 0.70 | 0.66 | 0.75 |
| 2022 | f | High | 70 | 18.71 | 19.37 | 19.32 | 19.41 | 0.65 | 0.61 | 0.70 |
| 2022 | f | High | 75 | 14.52 | 15.10 | 15.06 | 15.15 | 0.58 | 0.53 | 0.62 |
| 2022 | f | High | 80 | 10.69 | 11.13 | 11.08 | 11.17 | 0.44 | 0.40 | 0.49 |
| 2022 | f | High | 85 | 7.35 | 7.73 | 7.69 | 7.77 | 0.38 | 0.34 | 0.43 |
| 2022 | f | High | 90 | 4.86 | 5.04 | 5.02 | 5.07 | 0.18 | 0.15 | 0.20 |
| 2022 | f | High | 95 | 3.28 | 3.31 | 3.29 | 3.33 | 0.03 | 0.01 | 0.05 |
| 2022 | f | High | 100 | 1.77 | 2.70 | 2.59 | 2.81 | 0.92 | 0.81 | 1.03 |
| 2022 | f | Intermediate | 30 | 54.27 | 54.83 | 54.66 | 55.00 | 0.56 | 0.39 | 0.73 |
| 2022 | f | Intermediate | 35 | 49.38 | 50.00 | 49.87 | 50.13 | 0.62 | 0.49 | 0.75 |
| 2022 | f | Intermediate | 40 | 44.53 | 45.14 | 45.01 | 45.27 | 0.60 | 0.47 | 0.73 |
| 2022 | f | Intermediate | 45 | 39.76 | 40.33 | 40.20 | 40.46 | 0.57 | 0.44 | 0.70 |
| 2022 | f | Intermediate | 50 | 35.06 | 35.56 | 35.44 | 35.69 | 0.51 | 0.38 | 0.64 |
| 2022 | f | Intermediate | 55 | 30.42 | 30.96 | 30.83 | 31.09 | 0.54 | 0.41 | 0.67 |
| 2022 | f | Intermediate | 60 | 25.90 | 26.46 | 26.33 | 26.59 | 0.57 | 0.44 | 0.70 |
| 2022 | f | Intermediate | 65 | 21.52 | 22.09 | 21.96 | 22.23 | 0.58 | 0.44 | 0.71 |
| 2022 | f | Intermediate | 70 | 17.40 | 17.89 | 17.75 | 18.03 | 0.50 | 0.36 | 0.64 |
| 2022 | f | Intermediate | 75 | 13.45 | 13.89 | 13.74 | 14.03 | 0.43 | 0.29 | 0.58 |
| 2022 | f | Intermediate | 80 | 9.79 | 10.19 | 10.04 | 10.34 | 0.40 | 0.24 | 0.55 |
| 2022 | f | Intermediate | 85 | 6.72 | 7.08 | 6.90 | 7.27 | 0.36 | 0.17 | 0.55 |
| 2022 | f | Intermediate | 90 | 4.40 | 4.73 | 4.48 | 4.97 | 0.33 | 0.08 | 0.57 |
| 2022 | f | Intermediate | 95 | 2.84 | 3.36 | 2.73 | 3.98 | 0.52 | -0.11 | 1.15 |
| 2022 | f | Intermediate | 100 | 1.76 | 2.08 | 1.94 | 2.23 | 0.32 | 0.17 | 0.47 |
| 2022 | f | TOT | 30 | 54.29 | 55.11 | 55.08 | 55.15 | 0.82 | 0.79 | 0.85 |
| 2022 | f | TOT | 35 | 49.38 | 50.23 | 50.19 | 50.26 | 0.85 | 0.81 | 0.88 |
| 2022 | f | TOT | 40 | 44.53 | 45.35 | 45.32 | 45.38 | 0.82 | 0.79 | 0.85 |
| 2022 | f | TOT | 45 | 39.70 | 40.50 | 40.47 | 40.53 | 0.80 | 0.77 | 0.83 |
| 2022 | f | TOT | 50 | 34.95 | 35.70 | 35.66 | 35.73 | 0.75 | 0.71 | 0.78 |
| 2022 | f | TOT | 55 | 30.28 | 31.02 | 30.99 | 31.05 | 0.74 | 0.71 | 0.77 |
| 2022 | f | TOT | 60 | 25.72 | 26.45 | 26.42 | 26.48 | 0.73 | 0.70 | 0.76 |
| 2022 | f | TOT | 65 | 21.32 | 22.00 | 21.97 | 22.03 | 0.68 | 0.65 | 0.72 |
| 2022 | f | TOT | 70 | 17.18 | 17.75 | 17.72 | 17.78 | 0.57 | 0.54 | 0.60 |
| 2022 | f | TOT | 75 | 13.26 | 13.71 | 13.68 | 13.75 | 0.45 | 0.42 | 0.49 |
| 2022 | f | TOT | 80 | 9.62 | 10.02 | 9.99 | 10.06 | 0.40 | 0.37 | 0.44 |
| 2022 | f | TOT | 85 | 6.59 | 6.90 | 6.87 | 6.93 | 0.31 | 0.28 | 0.35 |
| 2022 | f | TOT | 90 | 4.31 | 4.56 | 4.53 | 4.59 | 0.25 | 0.22 | 0.28 |
| 2022 | f | TOT | 95 | 2.80 | 3.02 | 3.00 | 3.03 | 0.22 | 0.20 | 0.23 |
| 2022 | f | TOT | 100 | 1.71 | 1.98 | 1.98 | 1.98 | 0.27 | 0.27 | 0.27 |
| 2022 | m | Basic | 30 | 45.93 | 47.29 | 47.22 | 47.36 | 1.36 | 1.28 | 1.43 |
| 2022 | m | Basic | 35 | 41.27 | 42.71 | 42.63 | 42.78 | 1.44 | 1.36 | 1.51 |
| 2022 | m | Basic | 40 | 36.73 | 38.12 | 38.05 | 38.19 | 1.39 | 1.32 | 1.46 |
| 2022 | m | Basic | 45 | 32.27 | 33.64 | 33.57 | 33.71 | 1.37 | 1.30 | 1.44 |
| 2022 | m | Basic | 50 | 28.03 | 29.24 | 29.17 | 29.30 | 1.21 | 1.14 | 1.27 |
| 2022 | m | Basic | 55 | 23.92 | 25.15 | 25.09 | 25.21 | 1.23 | 1.16 | 1.29 |
| 2022 | m | Basic | 60 | 20.14 | 21.11 | 21.07 | 21.16 | 0.97 | 0.92 | 1.02 |
| 2022 | m | Basic | 65 | 16.56 | 17.57 | 17.52 | 17.62 | 1.01 | 0.96 | 1.06 |
| 2022 | m | Basic | 70 | 13.26 | 14.21 | 14.16 | 14.27 | 0.95 | 0.90 | 1.01 |
| 2022 | m | Basic | 75 | 10.24 | 11.03 | 10.97 | 11.09 | 0.78 | 0.72 | 0.84 |
| 2022 | m | Basic | 80 | 7.54 | 8.14 | 8.08 | 8.20 | 0.59 | 0.53 | 0.65 |
| 2022 | m | Basic | 85 | 5.22 | 5.67 | 5.62 | 5.71 | 0.45 | 0.40 | 0.49 |
| 2022 | m | Basic | 90 | 3.63 | 3.93 | 3.87 | 3.99 | 0.30 | 0.24 | 0.37 |
| 2022 | m | Basic | 95 | 2.90 | 2.68 | 2.64 | 2.72 | -0.22 | -0.26 | -0.18 |
| 2022 | m | Basic | 100 | 1.48 | 1.96 | 1.94 | 1.98 | 0.48 | 0.46 | 0.50 |
| 2022 | m | High | 30 | 53.14 | 54.07 | 54.03 | 54.11 | 0.93 | 0.89 | 0.98 |
| 2022 | m | High | 35 | 48.23 | 49.13 | 49.09 | 49.18 | 0.91 | 0.86 | 0.95 |
| 2022 | m | High | 40 | 43.33 | 44.25 | 44.21 | 44.30 | 0.92 | 0.87 | 0.96 |
| 2022 | m | High | 45 | 38.47 | 39.39 | 39.35 | 39.44 | 0.93 | 0.88 | 0.97 |
| 2022 | m | High | 50 | 33.71 | 34.56 | 34.52 | 34.61 | 0.85 | 0.81 | 0.90 |
| 2022 | m | High | 55 | 29.02 | 29.83 | 29.79 | 29.88 | 0.82 | 0.77 | 0.86 |
| 2022 | m | High | 60 | 24.50 | 25.31 | 25.27 | 25.35 | 0.81 | 0.76 | 0.85 |
| 2022 | m | High | 65 | 20.16 | 20.91 | 20.87 | 20.95 | 0.75 | 0.71 | 0.79 |
| 2022 | m | High | 70 | 16.07 | 16.68 | 16.64 | 16.72 | 0.62 | 0.58 | 0.66 |
| 2022 | m | High | 75 | 12.29 | 12.89 | 12.85 | 12.94 | 0.61 | 0.56 | 0.65 |
| 2022 | m | High | 80 | 8.90 | 9.40 | 9.36 | 9.45 | 0.50 | 0.45 | 0.54 |
| 2022 | m | High | 85 | 6.16 | 6.40 | 6.36 | 6.45 | 0.25 | 0.21 | 0.29 |
| 2022 | m | High | 90 | 3.92 | 3.98 | 3.95 | 4.01 | 0.06 | 0.03 | 0.10 |
| 2022 | m | High | 95 | 2.69 | 2.51 | 2.41 | 2.62 | -0.18 | -0.28 | -0.07 |
| 2022 | m | High | 100 | 1.57 | 3.72 | 3.52 | 3.91 | 2.14 | 1.94 | 2.34 |
| 2022 | m | Intermediate | 30 | 49.28 | 50.43 | 50.37 | 50.50 | 1.15 | 1.08 | 1.22 |
| 2022 | m | Intermediate | 35 | 44.50 | 45.62 | 45.55 | 45.68 | 1.12 | 1.05 | 1.19 |
| 2022 | m | Intermediate | 40 | 39.74 | 40.84 | 40.77 | 40.90 | 1.10 | 1.03 | 1.16 |
| 2022 | m | Intermediate | 45 | 35.05 | 36.12 | 36.05 | 36.18 | 1.07 | 1.01 | 1.14 |
| 2022 | m | Intermediate | 50 | 30.51 | 31.49 | 31.42 | 31.55 | 0.98 | 0.91 | 1.04 |
| 2022 | m | Intermediate | 55 | 26.11 | 26.98 | 26.92 | 27.05 | 0.88 | 0.81 | 0.94 |
| 2022 | m | Intermediate | 60 | 21.85 | 22.71 | 22.65 | 22.77 | 0.85 | 0.80 | 0.91 |
| 2022 | m | Intermediate | 65 | 17.96 | 18.74 | 18.69 | 18.80 | 0.78 | 0.72 | 0.83 |
| 2022 | m | Intermediate | 70 | 14.44 | 14.94 | 14.89 | 14.99 | 0.50 | 0.45 | 0.55 |
| 2022 | m | Intermediate | 75 | 11.09 | 11.55 | 11.50 | 11.60 | 0.46 | 0.41 | 0.51 |
| 2022 | m | Intermediate | 80 | 8.01 | 8.51 | 8.45 | 8.57 | 0.49 | 0.43 | 0.55 |
| 2022 | m | Intermediate | 85 | 5.62 | 5.75 | 5.69 | 5.81 | 0.13 | 0.07 | 0.19 |
| 2022 | m | Intermediate | 90 | 3.70 | 3.75 | 3.64 | 3.86 | 0.04 | -0.07 | 0.15 |
| 2022 | m | Intermediate | 95 | 2.63 | 3.07 | 2.93 | 3.21 | 0.44 | 0.30 | 0.59 |
| 2022 | m | Intermediate | 100 | 1.18 | 2.05 | 2.02 | 2.08 | 0.86 | 0.83 | 0.90 |
| 2022 | m | TOT | 30 | 49.52 | 50.72 | 50.69 | 50.76 | 1.20 | 1.17 | 1.24 |
| 2022 | m | TOT | 35 | 44.72 | 45.92 | 45.88 | 45.95 | 1.19 | 1.16 | 1.23 |
| 2022 | m | TOT | 40 | 39.96 | 41.14 | 41.10 | 41.18 | 1.18 | 1.14 | 1.21 |
| 2022 | m | TOT | 45 | 35.25 | 36.42 | 36.38 | 36.45 | 1.16 | 1.13 | 1.20 |
| 2022 | m | TOT | 50 | 30.68 | 31.75 | 31.71 | 31.78 | 1.07 | 1.03 | 1.10 |
| 2022 | m | TOT | 55 | 26.23 | 27.24 | 27.20 | 27.28 | 1.01 | 0.98 | 1.05 |
| 2022 | m | TOT | 60 | 21.97 | 22.92 | 22.89 | 22.96 | 0.95 | 0.92 | 0.98 |
| 2022 | m | TOT | 65 | 17.99 | 18.90 | 18.87 | 18.94 | 0.91 | 0.88 | 0.94 |
| 2022 | m | TOT | 70 | 14.35 | 15.08 | 15.05 | 15.11 | 0.74 | 0.70 | 0.77 |
| 2022 | m | TOT | 75 | 10.99 | 11.65 | 11.61 | 11.68 | 0.66 | 0.63 | 0.69 |
| 2022 | m | TOT | 80 | 7.96 | 8.53 | 8.50 | 8.56 | 0.56 | 0.53 | 0.60 |
| 2022 | m | TOT | 85 | 5.51 | 5.85 | 5.83 | 5.88 | 0.34 | 0.32 | 0.37 |
| 2022 | m | TOT | 90 | 3.72 | 3.91 | 3.89 | 3.93 | 0.19 | 0.17 | 0.21 |
| 2022 | m | TOT | 95 | 2.90 | 2.70 | 2.69 | 2.71 | -0.20 | -0.21 | -0.19 |
| 2022 | m | TOT | 100 | 1.45 | 2.18 | 2.17 | 2.19 | 0.72 | 0.71 | 0.73 |
| 2023 | f | Basic | 30 | 50.92 | 51.91 | 51.53 | 52.29 | 0.99 | 0.61 | 1.37 |
| 2023 | f | Basic | 35 | 46.17 | 47.12 | 46.85 | 47.39 | 0.95 | 0.68 | 1.21 |
| 2023 | f | Basic | 40 | 41.60 | 42.48 | 42.22 | 42.75 | 0.88 | 0.62 | 1.15 |
| 2023 | f | Basic | 45 | 36.93 | 37.91 | 37.66 | 38.16 | 0.98 | 0.73 | 1.23 |
| 2023 | f | Basic | 50 | 32.48 | 33.36 | 33.18 | 33.55 | 0.89 | 0.70 | 1.07 |
| 2023 | f | Basic | 55 | 28.30 | 29.15 | 28.96 | 29.33 | 0.84 | 0.66 | 1.03 |
| 2023 | f | Basic | 60 | 24.31 | 25.04 | 24.86 | 25.21 | 0.73 | 0.55 | 0.90 |
| 2023 | f | Basic | 65 | 20.38 | 20.99 | 20.81 | 21.16 | 0.61 | 0.43 | 0.78 |
| 2023 | f | Basic | 70 | 16.59 | 17.03 | 16.85 | 17.20 | 0.43 | 0.26 | 0.61 |
| 2023 | f | Basic | 75 | 12.97 | 13.20 | 13.01 | 13.38 | 0.23 | 0.04 | 0.41 |
| 2023 | f | Basic | 80 | 9.58 | 9.73 | 9.52 | 9.94 | 0.15 | -0.06 | 0.36 |
| 2023 | f | Basic | 85 | 6.68 | 6.72 | 6.50 | 6.94 | 0.04 | -0.17 | 0.26 |
| 2023 | f | Basic | 90 | 4.40 | 4.46 | 4.27 | 4.66 | 0.06 | -0.13 | 0.25 |
| 2023 | f | Basic | 95 | 2.91 | 2.91 | 2.88 | 2.94 | 0.00 | -0.03 | 0.03 |
| 2023 | f | Basic | 100 | 1.80 | 1.88 | 1.72 | 2.03 | 0.08 | -0.07 | 0.23 |
| 2023 | f | High | 30 | 57.04 | 57.74 | 57.69 | 57.79 | 0.70 | 0.65 | 0.76 |
| 2023 | f | High | 35 | 52.08 | 52.77 | 52.72 | 52.83 | 0.69 | 0.64 | 0.75 |
| 2023 | f | High | 40 | 47.14 | 47.83 | 47.77 | 47.88 | 0.69 | 0.64 | 0.74 |
| 2023 | f | High | 45 | 42.25 | 42.90 | 42.85 | 42.95 | 0.65 | 0.59 | 0.70 |
| 2023 | f | High | 50 | 37.39 | 38.04 | 37.98 | 38.09 | 0.64 | 0.59 | 0.70 |
| 2023 | f | High | 55 | 32.60 | 33.23 | 33.17 | 33.28 | 0.62 | 0.57 | 0.68 |
| 2023 | f | High | 60 | 27.92 | 28.51 | 28.46 | 28.56 | 0.59 | 0.53 | 0.64 |
| 2023 | f | High | 65 | 23.37 | 23.88 | 23.83 | 23.94 | 0.51 | 0.46 | 0.57 |
| 2023 | f | High | 70 | 18.90 | 19.45 | 19.39 | 19.50 | 0.54 | 0.49 | 0.60 |
| 2023 | f | High | 75 | 14.64 | 15.18 | 15.12 | 15.23 | 0.54 | 0.48 | 0.59 |
| 2023 | f | High | 80 | 10.82 | 11.19 | 11.13 | 11.24 | 0.37 | 0.31 | 0.42 |
| 2023 | f | High | 85 | 7.58 | 7.77 | 7.72 | 7.83 | 0.19 | 0.14 | 0.24 |
| 2023 | f | High | 90 | 4.65 | 5.06 | 5.03 | 5.09 | 0.41 | 0.38 | 0.44 |
| 2023 | f | High | 95 | 2.91 | 3.32 | 3.30 | 3.35 | 0.42 | 0.39 | 0.44 |
| 2023 | f | High | 100 | 2.05 | 2.80 | 2.66 | 2.93 | 0.75 | 0.62 | 0.89 |
| 2023 | f | Intermediate | 30 | 54.56 | 54.79 | 54.58 | 55.01 | 0.24 | 0.02 | 0.45 |
| 2023 | f | Intermediate | 35 | 49.68 | 49.93 | 49.77 | 50.09 | 0.25 | 0.09 | 0.41 |
| 2023 | f | Intermediate | 40 | 44.81 | 45.07 | 44.91 | 45.23 | 0.26 | 0.10 | 0.42 |
| 2023 | f | Intermediate | 45 | 40.05 | 40.27 | 40.11 | 40.43 | 0.22 | 0.06 | 0.38 |
| 2023 | f | Intermediate | 50 | 35.29 | 35.51 | 35.36 | 35.67 | 0.22 | 0.06 | 0.38 |
| 2023 | f | Intermediate | 55 | 30.65 | 30.91 | 30.75 | 31.06 | 0.26 | 0.10 | 0.41 |
| 2023 | f | Intermediate | 60 | 26.11 | 26.40 | 26.24 | 26.56 | 0.30 | 0.14 | 0.46 |
| 2023 | f | Intermediate | 65 | 21.74 | 22.03 | 21.87 | 22.20 | 0.29 | 0.13 | 0.46 |
| 2023 | f | Intermediate | 70 | 17.66 | 17.83 | 17.66 | 18.00 | 0.17 | 0.00 | 0.35 |
| 2023 | f | Intermediate | 75 | 13.72 | 13.82 | 13.63 | 14.00 | 0.09 | -0.09 | 0.28 |
| 2023 | f | Intermediate | 80 | 10.09 | 10.14 | 9.95 | 10.33 | 0.05 | -0.14 | 0.24 |
| 2023 | f | Intermediate | 85 | 7.01 | 7.03 | 6.80 | 7.26 | 0.03 | -0.21 | 0.26 |
| 2023 | f | Intermediate | 90 | 4.52 | 4.70 | 4.39 | 5.00 | 0.17 | -0.14 | 0.48 |
| 2023 | f | Intermediate | 95 | 2.87 | 3.24 | 2.45 | 4.02 | 0.37 | -0.41 | 1.15 |
| 2023 | f | Intermediate | 100 | 1.90 | 1.96 | 1.80 | 2.13 | 0.07 | -0.10 | 0.23 |
| 2023 | f | TOT | 30 | 54.69 | 55.20 | 55.16 | 55.24 | 0.51 | 0.47 | 0.55 |
| 2023 | f | TOT | 35 | 49.79 | 50.30 | 50.26 | 50.34 | 0.51 | 0.47 | 0.55 |
| 2023 | f | TOT | 40 | 44.92 | 45.42 | 45.38 | 45.46 | 0.50 | 0.46 | 0.54 |
| 2023 | f | TOT | 45 | 40.10 | 40.58 | 40.54 | 40.61 | 0.47 | 0.43 | 0.51 |
| 2023 | f | TOT | 50 | 35.31 | 35.77 | 35.73 | 35.81 | 0.46 | 0.42 | 0.50 |
| 2023 | f | TOT | 55 | 30.63 | 31.09 | 31.05 | 31.13 | 0.46 | 0.42 | 0.50 |
| 2023 | f | TOT | 60 | 26.07 | 26.51 | 26.48 | 26.55 | 0.45 | 0.41 | 0.49 |
| 2023 | f | TOT | 65 | 21.66 | 22.06 | 22.02 | 22.10 | 0.40 | 0.36 | 0.44 |
| 2023 | f | TOT | 70 | 17.47 | 17.80 | 17.76 | 17.84 | 0.33 | 0.29 | 0.37 |
| 2023 | f | TOT | 75 | 13.52 | 13.75 | 13.71 | 13.79 | 0.23 | 0.19 | 0.27 |
| 2023 | f | TOT | 80 | 9.92 | 10.06 | 10.02 | 10.10 | 0.14 | 0.10 | 0.18 |
| 2023 | f | TOT | 85 | 6.88 | 6.92 | 6.88 | 6.96 | 0.04 | 0.00 | 0.08 |
| 2023 | f | TOT | 90 | 4.46 | 4.57 | 4.54 | 4.60 | 0.11 | 0.08 | 0.14 |
| 2023 | f | TOT | 95 | 2.90 | 3.00 | 2.98 | 3.02 | 0.10 | 0.08 | 0.12 |
| 2023 | f | TOT | 100 | 1.84 | 1.96 | 1.96 | 1.96 | 0.12 | 0.12 | 0.12 |
| 2023 | m | Basic | 30 | 46.33 | 47.47 | 47.39 | 47.56 | 1.14 | 1.05 | 1.22 |
| 2023 | m | Basic | 35 | 41.67 | 42.88 | 42.80 | 42.97 | 1.21 | 1.13 | 1.30 |
| 2023 | m | Basic | 40 | 37.04 | 38.27 | 38.19 | 38.36 | 1.23 | 1.15 | 1.31 |
| 2023 | m | Basic | 45 | 32.61 | 33.78 | 33.70 | 33.86 | 1.17 | 1.09 | 1.25 |
| 2023 | m | Basic | 50 | 28.40 | 29.36 | 29.28 | 29.43 | 0.96 | 0.89 | 1.04 |
| 2023 | m | Basic | 55 | 24.31 | 25.26 | 25.19 | 25.33 | 0.95 | 0.87 | 1.02 |
| 2023 | m | Basic | 60 | 20.48 | 21.19 | 21.13 | 21.25 | 0.71 | 0.65 | 0.77 |
| 2023 | m | Basic | 65 | 16.89 | 17.65 | 17.59 | 17.71 | 0.76 | 0.70 | 0.82 |
| 2023 | m | Basic | 70 | 13.53 | 14.28 | 14.22 | 14.35 | 0.75 | 0.69 | 0.82 |
| 2023 | m | Basic | 75 | 10.46 | 11.09 | 11.02 | 11.17 | 0.64 | 0.57 | 0.71 |
| 2023 | m | Basic | 80 | 7.62 | 8.19 | 8.12 | 8.26 | 0.57 | 0.50 | 0.64 |
| 2023 | m | Basic | 85 | 5.31 | 5.69 | 5.64 | 5.75 | 0.38 | 0.33 | 0.44 |
| 2023 | m | Basic | 90 | 3.60 | 3.96 | 3.88 | 4.03 | 0.36 | 0.29 | 0.44 |
| 2023 | m | Basic | 95 | 2.60 | 2.69 | 2.64 | 2.74 | 0.09 | 0.04 | 0.13 |
| 2023 | m | Basic | 100 | 1.89 | 1.97 | 1.95 | 2.00 | 0.08 | 0.06 | 0.11 |
| 2023 | m | High | 30 | 53.55 | 54.30 | 54.25 | 54.35 | 0.75 | 0.70 | 0.81 |
| 2023 | m | High | 35 | 48.63 | 49.36 | 49.31 | 49.41 | 0.73 | 0.67 | 0.78 |
| 2023 | m | High | 40 | 43.74 | 44.48 | 44.43 | 44.53 | 0.74 | 0.69 | 0.79 |
| 2023 | m | High | 45 | 38.87 | 39.62 | 39.57 | 39.67 | 0.75 | 0.70 | 0.80 |
| 2023 | m | High | 50 | 34.03 | 34.78 | 34.73 | 34.83 | 0.75 | 0.69 | 0.80 |
| 2023 | m | High | 55 | 29.37 | 30.04 | 29.98 | 30.09 | 0.67 | 0.62 | 0.72 |
| 2023 | m | High | 60 | 24.83 | 25.51 | 25.45 | 25.56 | 0.68 | 0.62 | 0.73 |
| 2023 | m | High | 65 | 20.46 | 21.09 | 21.04 | 21.15 | 0.64 | 0.58 | 0.69 |
| 2023 | m | High | 70 | 16.32 | 16.84 | 16.80 | 16.89 | 0.52 | 0.47 | 0.57 |
| 2023 | m | High | 75 | 12.55 | 13.05 | 13.00 | 13.10 | 0.51 | 0.45 | 0.56 |
| 2023 | m | High | 80 | 9.13 | 9.55 | 9.50 | 9.61 | 0.42 | 0.37 | 0.48 |
| 2023 | m | High | 85 | 6.17 | 6.55 | 6.50 | 6.60 | 0.37 | 0.32 | 0.43 |
| 2023 | m | High | 90 | 4.04 | 4.16 | 4.11 | 4.20 | 0.12 | 0.07 | 0.16 |
| 2023 | m | High | 95 | 2.52 | 3.16 | 3.02 | 3.29 | 0.64 | 0.50 | 0.77 |
| 2023 | m | High | 100 | 2.02 | 4.05 | 3.81 | 4.30 | 2.03 | 1.79 | 2.28 |
| 2023 | m | Intermediate | 30 | 49.39 | 50.62 | 50.54 | 50.70 | 1.23 | 1.15 | 1.31 |
| 2023 | m | Intermediate | 35 | 44.64 | 45.79 | 45.71 | 45.87 | 1.16 | 1.08 | 1.24 |
| 2023 | m | Intermediate | 40 | 39.91 | 41.00 | 40.93 | 41.08 | 1.10 | 1.02 | 1.18 |
| 2023 | m | Intermediate | 45 | 35.25 | 36.28 | 36.20 | 36.36 | 1.03 | 0.95 | 1.11 |
| 2023 | m | Intermediate | 50 | 30.64 | 31.63 | 31.56 | 31.71 | 1.00 | 0.92 | 1.07 |
| 2023 | m | Intermediate | 55 | 26.24 | 27.11 | 27.03 | 27.18 | 0.86 | 0.79 | 0.94 |
| 2023 | m | Intermediate | 60 | 22.00 | 22.81 | 22.74 | 22.88 | 0.80 | 0.74 | 0.87 |
| 2023 | m | Intermediate | 65 | 18.10 | 18.82 | 18.75 | 18.88 | 0.71 | 0.65 | 0.78 |
| 2023 | m | Intermediate | 70 | 14.49 | 14.99 | 14.93 | 15.05 | 0.50 | 0.44 | 0.56 |
| 2023 | m | Intermediate | 75 | 11.14 | 11.58 | 11.52 | 11.65 | 0.45 | 0.39 | 0.51 |
| 2023 | m | Intermediate | 80 | 8.20 | 8.53 | 8.46 | 8.61 | 0.34 | 0.26 | 0.41 |
| 2023 | m | Intermediate | 85 | 5.87 | 5.74 | 5.67 | 5.82 | -0.12 | -0.19 | -0.05 |
| 2023 | m | Intermediate | 90 | 3.93 | 3.72 | 3.58 | 3.85 | -0.21 | -0.35 | -0.08 |
| 2023 | m | Intermediate | 95 | 2.65 | 3.13 | 2.96 | 3.30 | 0.48 | 0.30 | 0.65 |
| 2023 | m | Intermediate | 100 | 1.67 | 2.11 | 2.07 | 2.16 | 0.45 | 0.40 | 0.49 |
| 2023 | m | TOT | 30 | 49.83 | 50.94 | 50.89 | 50.98 | 1.11 | 1.06 | 1.15 |
| 2023 | m | TOT | 35 | 45.04 | 46.12 | 46.08 | 46.16 | 1.08 | 1.04 | 1.12 |
| 2023 | m | TOT | 40 | 40.28 | 41.34 | 41.30 | 41.38 | 1.06 | 1.01 | 1.10 |
| 2023 | m | TOT | 45 | 35.60 | 36.61 | 36.57 | 36.66 | 1.02 | 0.97 | 1.06 |
| 2023 | m | TOT | 50 | 30.97 | 31.93 | 31.89 | 31.98 | 0.96 | 0.92 | 1.01 |
| 2023 | m | TOT | 55 | 26.53 | 27.41 | 27.37 | 27.45 | 0.88 | 0.83 | 0.92 |
| 2023 | m | TOT | 60 | 22.26 | 23.07 | 23.03 | 23.11 | 0.81 | 0.77 | 0.85 |
| 2023 | m | TOT | 65 | 18.27 | 19.03 | 18.99 | 19.07 | 0.76 | 0.72 | 0.80 |
| 2023 | m | TOT | 70 | 14.55 | 15.18 | 15.14 | 15.22 | 0.63 | 0.59 | 0.67 |
| 2023 | m | TOT | 75 | 11.16 | 11.73 | 11.69 | 11.77 | 0.57 | 0.53 | 0.61 |
| 2023 | m | TOT | 80 | 8.11 | 8.60 | 8.56 | 8.64 | 0.49 | 0.45 | 0.53 |
| 2023 | m | TOT | 85 | 5.61 | 5.88 | 5.86 | 5.91 | 0.28 | 0.25 | 0.30 |
| 2023 | m | TOT | 90 | 3.74 | 3.92 | 3.90 | 3.94 | 0.17 | 0.15 | 0.20 |
| 2023 | m | TOT | 95 | 2.59 | 2.71 | 2.70 | 2.72 | 0.12 | 0.11 | 0.13 |
| 2023 | m | TOT | 100 | 1.89 | 2.24 | 2.22 | 2.25 | 0.34 | 0.33 | 0.36 |
| 2024 | f | Basic | 30 | 51.35 | 51.92 | 51.42 | 52.41 | 0.56 | 0.07 | 1.06 |
| 2024 | f | Basic | 35 | 46.66 | 47.11 | 46.79 | 47.43 | 0.45 | 0.13 | 0.78 |
| 2024 | f | Basic | 40 | 41.97 | 42.48 | 42.16 | 42.80 | 0.51 | 0.19 | 0.83 |
| 2024 | f | Basic | 45 | 37.42 | 37.92 | 37.62 | 38.22 | 0.50 | 0.19 | 0.80 |
| 2024 | f | Basic | 50 | 32.95 | 33.36 | 33.14 | 33.57 | 0.41 | 0.20 | 0.62 |
| 2024 | f | Basic | 55 | 28.69 | 29.14 | 28.92 | 29.36 | 0.44 | 0.22 | 0.66 |
| 2024 | f | Basic | 60 | 24.66 | 25.04 | 24.84 | 25.25 | 0.38 | 0.17 | 0.58 |
| 2024 | f | Basic | 65 | 20.74 | 21.00 | 20.80 | 21.20 | 0.26 | 0.06 | 0.46 |
| 2024 | f | Basic | 70 | 16.88 | 17.05 | 16.84 | 17.25 | 0.17 | -0.03 | 0.37 |
| 2024 | f | Basic | 75 | 13.26 | 13.22 | 13.01 | 13.44 | -0.04 | -0.25 | 0.18 |
| 2024 | f | Basic | 80 | 9.89 | 9.76 | 9.51 | 10.00 | -0.13 | -0.37 | 0.11 |
| 2024 | f | Basic | 85 | 6.97 | 6.74 | 6.49 | 7.00 | -0.23 | -0.48 | 0.02 |
| 2024 | f | Basic | 90 | 4.64 | 4.48 | 4.25 | 4.70 | -0.16 | -0.38 | 0.06 |
| 2024 | f | Basic | 95 | 3.02 | 2.91 | 2.88 | 2.94 | -0.11 | -0.15 | -0.08 |
| 2024 | f | Basic | 100 | 1.95 | 1.86 | 1.68 | 2.03 | -0.09 | -0.26 | 0.08 |
| 2024 | f | High | 30 | 57.61 | 57.86 | 57.80 | 57.92 | 0.25 | 0.19 | 0.31 |
| 2024 | f | High | 35 | 52.65 | 52.89 | 52.83 | 52.95 | 0.24 | 0.18 | 0.30 |
| 2024 | f | High | 40 | 47.73 | 47.94 | 47.88 | 48.01 | 0.22 | 0.16 | 0.28 |
| 2024 | f | High | 45 | 42.83 | 43.01 | 42.95 | 43.07 | 0.18 | 0.12 | 0.25 |
| 2024 | f | High | 50 | 37.96 | 38.14 | 38.08 | 38.21 | 0.18 | 0.12 | 0.25 |
| 2024 | f | High | 55 | 33.19 | 33.33 | 33.27 | 33.39 | 0.14 | 0.08 | 0.20 |
| 2024 | f | High | 60 | 28.51 | 28.61 | 28.54 | 28.67 | 0.10 | 0.03 | 0.16 |
| 2024 | f | High | 65 | 23.91 | 23.97 | 23.91 | 24.03 | 0.06 | 0.00 | 0.12 |
| 2024 | f | High | 70 | 19.49 | 19.53 | 19.47 | 19.59 | 0.04 | -0.02 | 0.10 |
| 2024 | f | High | 75 | 15.27 | 15.25 | 15.19 | 15.31 | -0.02 | -0.08 | 0.04 |
| 2024 | f | High | 80 | 11.37 | 11.25 | 11.19 | 11.31 | -0.12 | -0.18 | -0.06 |
| 2024 | f | High | 85 | 7.86 | 7.82 | 7.76 | 7.88 | -0.04 | -0.10 | 0.02 |
| 2024 | f | High | 90 | 5.11 | 5.08 | 5.04 | 5.11 | -0.03 | -0.07 | 0.00 |
| 2024 | f | High | 95 | 3.45 | 3.33 | 3.30 | 3.36 | -0.12 | -0.15 | -0.09 |
| 2024 | f | High | 100 | 2.67 | 2.91 | 2.75 | 3.07 | 0.24 | 0.08 | 0.40 |
| 2024 | f | Intermediate | 30 | 55.37 | 54.75 | 54.50 | 55.01 | -0.62 | -0.88 | -0.36 |
| 2024 | f | Intermediate | 35 | 50.47 | 49.87 | 49.68 | 50.07 | -0.60 | -0.79 | -0.40 |
| 2024 | f | Intermediate | 40 | 45.60 | 45.01 | 44.81 | 45.20 | -0.59 | -0.78 | -0.39 |
| 2024 | f | Intermediate | 45 | 40.78 | 40.21 | 40.01 | 40.40 | -0.57 | -0.77 | -0.38 |
| 2024 | f | Intermediate | 50 | 36.01 | 35.47 | 35.28 | 35.66 | -0.54 | -0.73 | -0.35 |
| 2024 | f | Intermediate | 55 | 31.33 | 30.86 | 30.66 | 31.05 | -0.48 | -0.67 | -0.28 |
| 2024 | f | Intermediate | 60 | 26.78 | 26.35 | 26.15 | 26.54 | -0.43 | -0.63 | -0.24 |
| 2024 | f | Intermediate | 65 | 22.39 | 21.98 | 21.78 | 22.18 | -0.41 | -0.61 | -0.21 |
| 2024 | f | Intermediate | 70 | 18.19 | 17.77 | 17.57 | 17.98 | -0.41 | -0.62 | -0.20 |
| 2024 | f | Intermediate | 75 | 14.13 | 13.75 | 13.53 | 13.97 | -0.38 | -0.60 | -0.16 |
| 2024 | f | Intermediate | 80 | 10.46 | 10.09 | 9.87 | 10.32 | -0.37 | -0.60 | -0.14 |
| 2024 | f | Intermediate | 85 | 7.46 | 6.99 | 6.70 | 7.27 | -0.48 | -0.76 | -0.19 |
| 2024 | f | Intermediate | 90 | 5.00 | 4.68 | 4.29 | 5.06 | -0.32 | -0.71 | 0.06 |
| 2024 | f | Intermediate | 95 | 3.15 | 3.15 | 2.17 | 4.13 | 0.00 | -0.98 | 0.98 |
| 2024 | f | Intermediate | 100 | 1.75 | 2.03 | 1.60 | 2.45 | 0.28 | -0.14 | 0.71 |
| 2024 | f | TOT | 30 | 55.30 | 55.28 | 55.24 | 55.32 | -0.02 | -0.06 | 0.03 |
| 2024 | f | TOT | 35 | 50.40 | 50.37 | 50.32 | 50.41 | -0.03 | -0.07 | 0.02 |
| 2024 | f | TOT | 40 | 45.52 | 45.49 | 45.45 | 45.54 | -0.03 | -0.07 | 0.02 |
| 2024 | f | TOT | 45 | 40.69 | 40.65 | 40.60 | 40.69 | -0.04 | -0.09 | 0.00 |
| 2024 | f | TOT | 50 | 35.89 | 35.85 | 35.80 | 35.89 | -0.04 | -0.08 | 0.01 |
| 2024 | f | TOT | 55 | 31.19 | 31.16 | 31.12 | 31.20 | -0.03 | -0.08 | 0.01 |
| 2024 | f | TOT | 60 | 26.62 | 26.58 | 26.54 | 26.62 | -0.04 | -0.09 | 0.00 |
| 2024 | f | TOT | 65 | 22.18 | 22.12 | 22.07 | 22.16 | -0.06 | -0.11 | -0.02 |
| 2024 | f | TOT | 70 | 17.93 | 17.85 | 17.80 | 17.89 | -0.09 | -0.13 | -0.04 |
| 2024 | f | TOT | 75 | 13.93 | 13.79 | 13.74 | 13.83 | -0.14 | -0.19 | -0.10 |
| 2024 | f | TOT | 80 | 10.30 | 10.10 | 10.05 | 10.15 | -0.20 | -0.25 | -0.15 |
| 2024 | f | TOT | 85 | 7.21 | 6.95 | 6.90 | 6.99 | -0.26 | -0.31 | -0.21 |
| 2024 | f | TOT | 90 | 4.76 | 4.58 | 4.54 | 4.62 | -0.18 | -0.21 | -0.14 |
| 2024 | f | TOT | 95 | 3.09 | 2.99 | 2.97 | 3.01 | -0.10 | -0.12 | -0.08 |
| 2024 | f | TOT | 100 | 1.98 | 1.97 | 1.97 | 1.98 | -0.01 | -0.01 | -0.01 |
| 2024 | m | Basic | 30 | 47.06 | 47.65 | 47.55 | 47.75 | 0.59 | 0.49 | 0.69 |
| 2024 | m | Basic | 35 | 42.38 | 43.05 | 42.96 | 43.15 | 0.67 | 0.58 | 0.77 |
| 2024 | m | Basic | 40 | 37.76 | 38.43 | 38.34 | 38.53 | 0.67 | 0.57 | 0.76 |
| 2024 | m | Basic | 45 | 33.33 | 33.92 | 33.83 | 34.02 | 0.59 | 0.50 | 0.69 |
| 2024 | m | Basic | 50 | 29.15 | 29.48 | 29.39 | 29.57 | 0.33 | 0.25 | 0.42 |
| 2024 | m | Basic | 55 | 25.08 | 25.37 | 25.28 | 25.45 | 0.29 | 0.20 | 0.37 |
| 2024 | m | Basic | 60 | 21.13 | 21.27 | 21.20 | 21.34 | 0.14 | 0.07 | 0.21 |
| 2024 | m | Basic | 65 | 17.44 | 17.73 | 17.66 | 17.80 | 0.29 | 0.22 | 0.36 |
| 2024 | m | Basic | 70 | 14.09 | 14.36 | 14.28 | 14.43 | 0.27 | 0.19 | 0.34 |
| 2024 | m | Basic | 75 | 11.06 | 11.16 | 11.08 | 11.24 | 0.10 | 0.01 | 0.18 |
| 2024 | m | Basic | 80 | 8.18 | 8.24 | 8.16 | 8.32 | 0.06 | -0.03 | 0.14 |
| 2024 | m | Basic | 85 | 5.75 | 5.72 | 5.66 | 5.79 | -0.03 | -0.09 | 0.04 |
| 2024 | m | Basic | 90 | 3.92 | 3.98 | 3.89 | 4.07 | 0.06 | -0.03 | 0.15 |
| 2024 | m | Basic | 95 | 2.76 | 2.70 | 2.64 | 2.76 | -0.06 | -0.12 | 0.00 |
| 2024 | m | Basic | 100 | 1.66 | 1.99 | 1.96 | 2.02 | 0.33 | 0.30 | 0.36 |
| 2024 | m | High | 30 | 54.00 | 54.47 | 54.40 | 54.53 | 0.47 | 0.40 | 0.53 |
| 2024 | m | High | 35 | 49.08 | 49.52 | 49.46 | 49.58 | 0.44 | 0.38 | 0.50 |
| 2024 | m | High | 40 | 44.17 | 44.65 | 44.58 | 44.71 | 0.47 | 0.41 | 0.53 |
| 2024 | m | High | 45 | 39.33 | 39.78 | 39.72 | 39.84 | 0.45 | 0.39 | 0.51 |
| 2024 | m | High | 50 | 34.54 | 34.93 | 34.87 | 34.99 | 0.39 | 0.33 | 0.45 |
| 2024 | m | High | 55 | 29.81 | 30.18 | 30.12 | 30.24 | 0.37 | 0.31 | 0.43 |
| 2024 | m | High | 60 | 25.22 | 25.64 | 25.58 | 25.70 | 0.41 | 0.35 | 0.47 |
| 2024 | m | High | 65 | 20.85 | 21.21 | 21.15 | 21.27 | 0.36 | 0.30 | 0.42 |
| 2024 | m | High | 70 | 16.76 | 16.94 | 16.88 | 16.99 | 0.17 | 0.12 | 0.23 |
| 2024 | m | High | 75 | 12.96 | 13.14 | 13.08 | 13.20 | 0.18 | 0.12 | 0.24 |
| 2024 | m | High | 80 | 9.47 | 9.62 | 9.56 | 9.68 | 0.15 | 0.09 | 0.21 |
| 2024 | m | High | 85 | 6.65 | 6.58 | 6.53 | 6.64 | -0.06 | -0.12 | 0.00 |
| 2024 | m | High | 90 | 4.39 | 4.16 | 4.10 | 4.21 | -0.23 | -0.29 | -0.18 |
| 2024 | m | High | 95 | 2.94 | 3.19 | 3.02 | 3.36 | 0.25 | 0.08 | 0.42 |
| 2024 | m | High | 100 | 1.70 | 4.42 | 4.13 | 4.72 | 2.73 | 2.43 | 3.02 |
| 2024 | m | Intermediate | 30 | 50.03 | 50.80 | 50.71 | 50.89 | 0.77 | 0.68 | 0.86 |
| 2024 | m | Intermediate | 35 | 45.28 | 45.97 | 45.88 | 46.06 | 0.68 | 0.59 | 0.78 |
| 2024 | m | Intermediate | 40 | 40.51 | 41.17 | 41.08 | 41.26 | 0.66 | 0.57 | 0.75 |
| 2024 | m | Intermediate | 45 | 35.87 | 36.43 | 36.34 | 36.52 | 0.57 | 0.48 | 0.66 |
| 2024 | m | Intermediate | 50 | 31.30 | 31.78 | 31.69 | 31.87 | 0.48 | 0.39 | 0.57 |
| 2024 | m | Intermediate | 55 | 26.79 | 27.23 | 27.14 | 27.31 | 0.44 | 0.36 | 0.53 |
| 2024 | m | Intermediate | 60 | 22.52 | 22.90 | 22.82 | 22.98 | 0.38 | 0.30 | 0.46 |
| 2024 | m | Intermediate | 65 | 18.55 | 18.89 | 18.82 | 18.97 | 0.34 | 0.27 | 0.42 |
| 2024 | m | Intermediate | 70 | 14.95 | 15.04 | 14.97 | 15.11 | 0.09 | 0.02 | 0.16 |
| 2024 | m | Intermediate | 75 | 11.60 | 11.62 | 11.55 | 11.69 | 0.02 | -0.05 | 0.09 |
| 2024 | m | Intermediate | 80 | 8.67 | 8.56 | 8.48 | 8.65 | -0.11 | -0.20 | -0.03 |
| 2024 | m | Intermediate | 85 | 6.23 | 5.73 | 5.65 | 5.82 | -0.49 | -0.58 | -0.41 |
| 2024 | m | Intermediate | 90 | 4.50 | 3.69 | 3.52 | 3.85 | -0.82 | -0.98 | -0.65 |
| 2024 | m | Intermediate | 95 | 3.13 | 3.19 | 2.99 | 3.39 | 0.06 | -0.14 | 0.25 |
| 2024 | m | Intermediate | 100 | 1.91 | 2.18 | 2.13 | 2.23 | 0.28 | 0.23 | 0.33 |
| 2024 | m | TOT | 30 | 50.49 | 51.15 | 51.10 | 51.20 | 0.66 | 0.61 | 0.70 |
| 2024 | m | TOT | 35 | 45.70 | 46.33 | 46.28 | 46.38 | 0.62 | 0.57 | 0.67 |
| 2024 | m | TOT | 40 | 40.92 | 41.54 | 41.49 | 41.59 | 0.62 | 0.57 | 0.67 |
| 2024 | m | TOT | 45 | 36.25 | 36.81 | 36.76 | 36.86 | 0.56 | 0.51 | 0.60 |
| 2024 | m | TOT | 50 | 31.66 | 32.11 | 32.07 | 32.16 | 0.45 | 0.40 | 0.50 |
| 2024 | m | TOT | 55 | 27.16 | 27.57 | 27.53 | 27.62 | 0.42 | 0.37 | 0.46 |
| 2024 | m | TOT | 60 | 22.84 | 23.21 | 23.17 | 23.26 | 0.38 | 0.33 | 0.42 |
| 2024 | m | TOT | 65 | 18.78 | 19.15 | 19.11 | 19.20 | 0.37 | 0.33 | 0.41 |
| 2024 | m | TOT | 70 | 15.08 | 15.28 | 15.23 | 15.32 | 0.19 | 0.15 | 0.24 |
| 2024 | m | TOT | 75 | 11.69 | 11.81 | 11.77 | 11.86 | 0.12 | 0.07 | 0.16 |
| 2024 | m | TOT | 80 | 8.61 | 8.67 | 8.63 | 8.71 | 0.06 | 0.02 | 0.10 |
| 2024 | m | TOT | 85 | 6.04 | 5.91 | 5.88 | 5.94 | -0.13 | -0.16 | -0.10 |
| 2024 | m | TOT | 90 | 4.11 | 3.92 | 3.90 | 3.95 | -0.19 | -0.22 | -0.16 |
| 2024 | m | TOT | 95 | 2.85 | 2.72 | 2.71 | 2.73 | -0.13 | -0.14 | -0.11 |
| 2024 | m | TOT | 100 | 1.70 | 2.29 | 2.28 | 2.31 | 0.60 | 0.58 | 0.61 |
